# Supplementary material for: Cooperative Fe sites on transition metal (oxy)hydroxides drive high oxygen evolution activity in base
Source: Nat Commun. 2023 Nov 24;14:7688. doi: 10.1038/s41467-023-43305-z (PMC10673886; doi:10.1038/s41467-023-43305-z)
Supplement: Supplementary file 1 — Supplementary Information [file 41467_2023_43305_MOESM1_ESM.pdf]

Supplementary Information

Cooperative Fe Sites on Transition Metal (Oxy)hydroxides Drive High Oxygen Evolution Activity in Base

Yingqing Ou[1,2ǂ], Liam P. Twight[1ǂ], Bipasa Samanta[3ǂ], Lu Liu[1,4], Santu Biswas,[3] Jessica L. Fehrs[1], Nicole A. Sagui[1], Javier Villalobos[5], Joaquín Morales-Santelices[5], Denis Antipin[5], Marcel Risch[5], Maytal Caspary Toroker[3,6]*, and Shannon W. Boettcher[1]*

[1] Department of Chemistry and Biochemistry and the Oregon Center for Electrochemistry, University of Oregon, Eugene, Oregon 97403, United States

[2] School of Chemistry and Chemical Engineering, Chongqing University, Chongqing 400044, China

[3] Department of Materials Science and Engineering, Technion—Israel Institute of Technology, Haifa 3200003, Israel

[4] School of Materials Science and Engineering, Chongqing University, Chongqing 400044, China

[5] Nachwuchsgruppe Gestaltung des Sauerstoffentwicklungsmechanismus, Helmholtz-Zentrum Berlin für Materialien und Energie, Hahn-Meitner-Platz 1, 14109 Berlin, Germany

[6] The Nancy and Stephen Grand Technion Energy Program, Haifa, Israel

e-mail:

* Shannon W. Boettcher: swb@uoregon.edu

* Maytal Caspary Toroker: maytalc@technion.ac.il

ǂ These authors contributed equally to the manuscript.

X-ray absorption spectroscopy measurements and methods.

XAS measurements at the Fe-K edge were carried out at the KMC-3 beamline[1] *using the CryoEXAFS endstation*[2] *at the BESSY II electron storage ring operated by the Helmholtz-Zentrum Berlin für Materialien und Energie.* The spectra were recorded in fluorescence mode using a 13-element Si drift detector from RaySpec. The monochromator was a double-crystal Si (111), and the polarization of the beam was horizontal. To obtain Fe K-edge spectra, a servo system (PID controller) was needed to subtract counts coming from other elements that absorb at the same energy. A home-made 3D printed XAS cell was used. X-rays can enter the cell through a Kapton window (5 mm diameter and 65 µm thick) and an electrolyte flow channel of 250 µm thick between this window and the sample, which was exposed to the electrolyte 5.0 mm in length and 7.0 mm in width. A Hg/HgO reference electrode (ALS Inc.) was placed before the inlet and the counter electrode was a Pt tube placed at the outlet. The 0.1 M KOH electrolyte was flown at 2.00 mL min^{-1} using a Fluigent FlowEZ system and controlled by a flow rate sensor (Flow EZ XL).

The data was collected to k = 13 $Å^{-1}$ and the energy was calibrated by assigning the inflection point of a Fe metal foil to 7112 eV.[3] The foil was measured immediately before the samples and re-measured periodically. All spectra were normalized by subtracting a constant obtained by fitting the data before the *K* edge and division by a 2nd polynomial function obtained by fitting the data after the *K* edge for XANES analysis. For EXAFS analysis, the data after the edge was instead fit by a knot-spline with 7-10 knots followed by subtraction of one. The procedure of data extraction is detailed elsewhere[4]. Glitches were removed from the data, for which the deviation of individual scans from the average of all scans was considered. The FT of the EXAFS was calculated between 22 and 244 eV (2.4 – 8.0 $Å^{-1}$) above the Fe-*K* edge (E_0 = 7112 eV). The extracted EXAFS was re-binned to give an equal step size of 0.05 $Å^{-1}$ in k-space. A cosine window covering 10% on the left side and 20% on the right side of the EXAFS spectra was used to suppress the side lobes in the FTs. Weighing of the intensity by k^2 was chosen to emphasize the oscillations between 2 and 8 $Å^{-1}$.

EXAFS simulations were performed using the software SimXLite (developed by Dr. Petko Chernev). After calculating the phase functions with FEFF8-Lite[5] version 8.5.3, self-consistent field option activated), atomic coordinates of the FEFF input files were generated from the structure of g-FeOOH (lepidocrocite)[6] [data: http://www.crystallography.net/cod/1538421.html]. An amplitude reduction factor (S_0^2) of 0.8 and energy shift DE$_0$ of 4.0 eV were used. They were determined by fits to g-FeOOH [data https://doi.org/10.48505/nims.2040] in an identical range as used for the samples. The data range used in the simulation was 49.4–231.8 eV (3.6–7.8 $Å^{-1}$) above the Fe-*K* edge (E_0 = 7112 eV) if not stated otherwise. The EXAFS simulations were performed

in(unfiltered) k-space and optimized by minimizing the sum of the squared deviations (error sum) between measured and simulated values using a least-squares fit. The fit was performed using the Levenberg–Marquardt algorithm with numerical derivatives. The Fe-O (reduced distance ~1.5 Å) and Fe-Fe (reduced distance ~2.6 Å) peaks in the FT were considered in the fit. The parameter errors were obtained as described in detail in Risch *et al.*[7] The essential steps are: (1) Fourier isolation of the data and fit between 0.00 and 3.2 Å (i.e., the first two peaks in the FT); (2) back transformation to k-space; (3) assumption that the minimized reduced error sum of the Fourier-filtered fit (given by steps 1-2) provides a sound estimate of the measurement error; (4) calculation of the parameter variances from the covariance matrix of the minimized fit parameters; (5) the 1s-standard deviation is obtained from the square root of the diagonal elements of the covariance matrix. The goodness of fit parameter, R_f, was obtained by summing the squared difference between data and fit divided by the squared data for each measured point; we provide it for data Fourier filtered between 0.00 and 3.2 Å (R_f').

Supplementary Discussion on the lack of redox behavior observable for Fe species.

From the Pourbaix diagram for Fe, Fe^{3+} has a wide range of redox stability. The higher valent states can be accessed only in a region of OER current, for example likely leading to the soluble species FeO_4^{2-} (Fe^{6+} formally, this degrades to release O_2 and has much positive charge on the O ligand compared to formal O^{2-}) and FeO_2^{2-} (formally Fe^{4+}). Hunter and Gray have shown that in non-aqueous electrolytes redox signatures related to the formation of such a species can be seen that are not present in water.[8]

The $Fe^{2/3+}$ couple can be accessed at lower potentials, but this is made difficult to quantify because the entire host Ni- or Co hydroxide is electrically an insulator at these potentials, isolating the Fe sites electronically. In previous work[9] we showed from *in-situ* in-plane conductivity measurements that the onset of conductivity for cobalt and nickel hydroxides is concurrent with the cobalt and nickel redox wave which is much higher potentials than one would expect the $Fe^{2/3+}$ couple to be. Previous work studying thin films of FeOOH have discussed this redox behavior[10].

Supplementary Discussion on the differences in Fe incorporation in NiOOH and CoOOH.

Co oxyhydroxides/hydroxides have stronger bonding than those of Ni. We have shown in AFM studies[11] of NiOOH and CoOOH nanosheets that the former undergoes much more dynamic

morphology changes than the latter. Specifically, NiOOH seems to restructure dramatically by dissolution and redeposition which is a process we expect would drive the dissolution of surface adsorbed Fe and then substitution of this dissolved Fe in Ni sites. This is consistent with empirically measured metal-hydroxide bond strengths[12] and metal oxide dissociation energies[13].

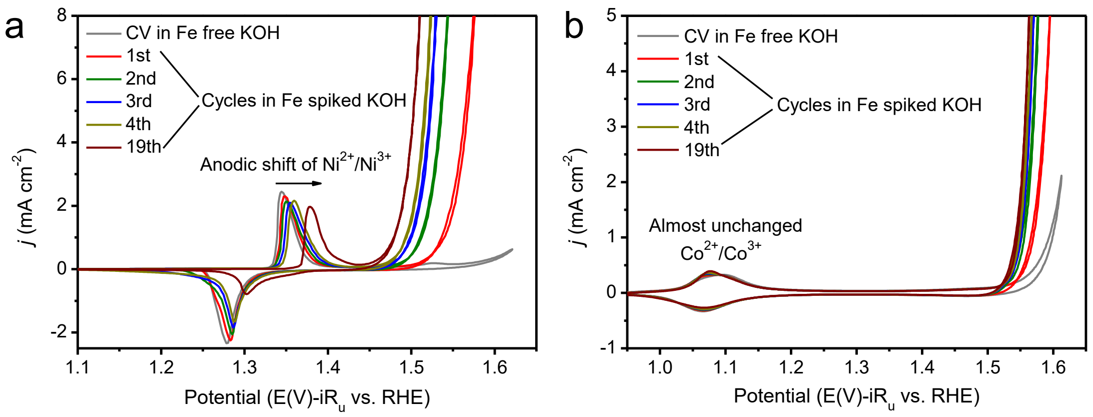

Supplementary Figure 1 | Cyclic voltammetry showing the effect of incorporated Fe from the electrolyte on the OER activity and redox profiles of **(a)** NiO_xH_y and **(b)** CoO_xH_y films. For the NiO_xH_y film, the first CV cycle after the addition of 0.1 ppm aq. Fe^{3+} into 1 M KOH resulted in ~87 mV decrease in overpotential (at 0.5 mA cm^{-2}), while the redox peak potentials of Ni^{2+}/Ni^{3+} shifted positive by ~4 mV. After the initial 4 cycles, less-pronounced activity enhancement was observed in the following cycles, while the redox peaks kept shifting anodic and shrinking in integrated peak area. For CoO_xH_y film, most of the activity increase also occurred in the initial 4 cycles, but the Co^{2+}/Co^{3+} redox peak potential and area were almost unaffected by Fe incorporation.

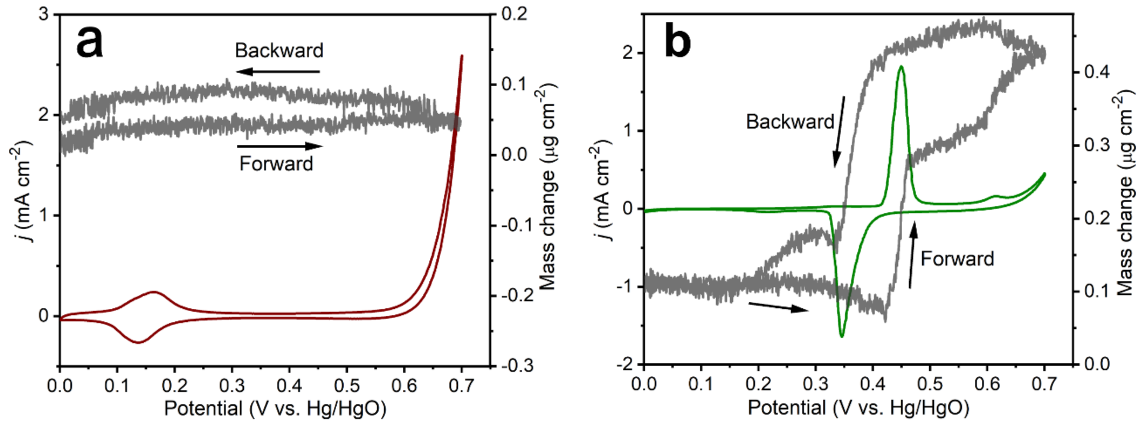

Supplementary Figure 2 | The *in-situ* mass change of freshly electrodeposited CoO_xH_y (**a**) and NiO_xH_y (**b**) films on the electrochemical quartz crystal microbalance substrate during the second voltammetry cycle in purified 1 M KOH solution.

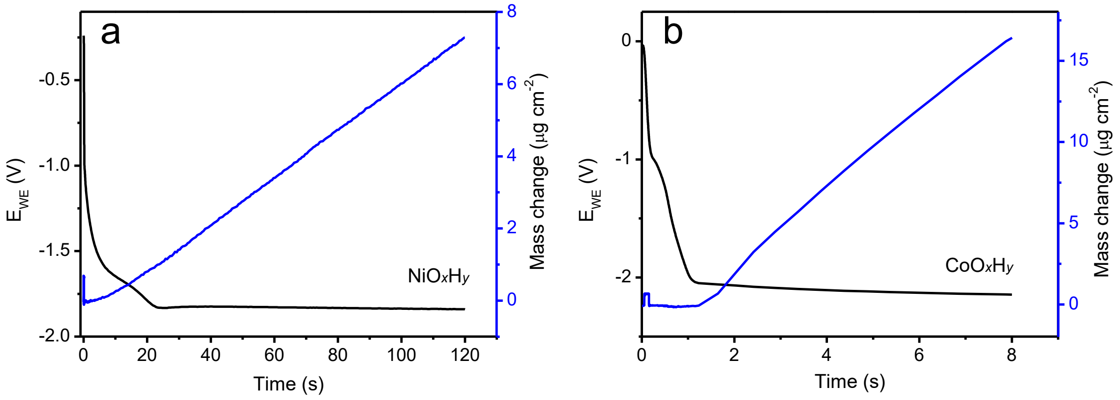

Supplementary Figure 3 | Potential vs. time deposition curves (black) and corresponding mass change profiles (blue) of EQCM electrodes for **(a)** NiO_xH_y film deposited at -0.1 mA cm^{-2} for 120 s, and **(b)** CoO_xH_y film deposited at -2 mA cm^{-2} for 8 s. The typical film mass loading for NiO_xH_y and CoO_xH_y are ~7.3 and 16.5 μg cm^{-2}, respectively.

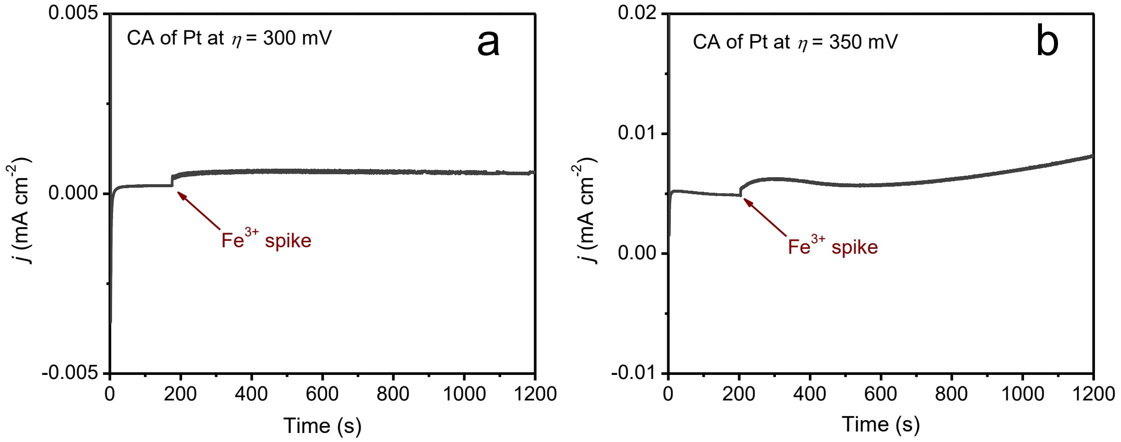

Supplementary Fig. 4 | The effect of Fe spiking on the OER activity of a Pt substrate under chronoamperometry measurements at overpotentials of **(a)** 300 mV and **(b)** 350 mV. It was found that the addition of Fe^{3+} (0.1 ppm) into 1 M KOH electrolyte caused negligible OER current increase for the Pt substrate. Thus, the dramatic current increase observed for NiO_xH_y or CoO_xH_y loaded Pt electrode was attributed to the adsorbed Fe species on (oxy)hydroxides rather than on underlying Pt substrate.

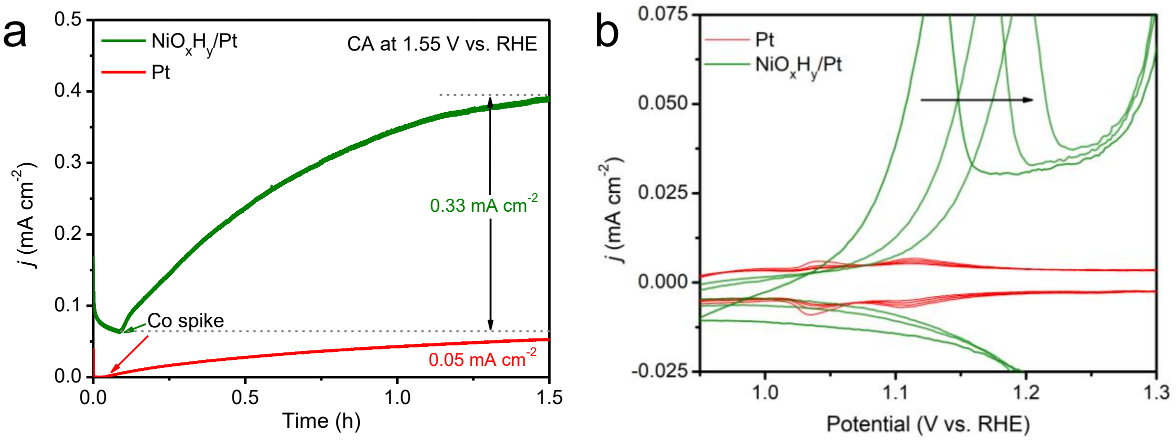

Supplementary Fig. 5 | (a) Chronoamperometry (CA) of bare Pt (red) and NiO_xH_y/Pt (green) electrodes in 0.1 ppm Co^{2+} spiked 1 M KOH solution. In the case of bare Pt substrate, the addition of Co^{2+} into the electrolyte resulted in much smaller current increase compared to NiO_xH_y/Pt. Thus, the current increase of NiO_xH_y/Pt mainly originated from adsorbed Co (oxy)hydroxides on the surface of NiO_xH_y film rather than on the Pt substrate. This could be explained by the substantially larger surface area of the porous NiO_xH_y film relative to the planar Pt substrate. **(b)** Voltammetry cycles after the Co^{2+} spiking CA measurement demonstrating the redox peaks of Co^{2+}/Co^{3+} on bare Pt (red) and NiO_xH_y/Pt (green) electrode. The integrated peak area on the Pt substrate is negligible compared to that on NiO_xH_y/Pt.

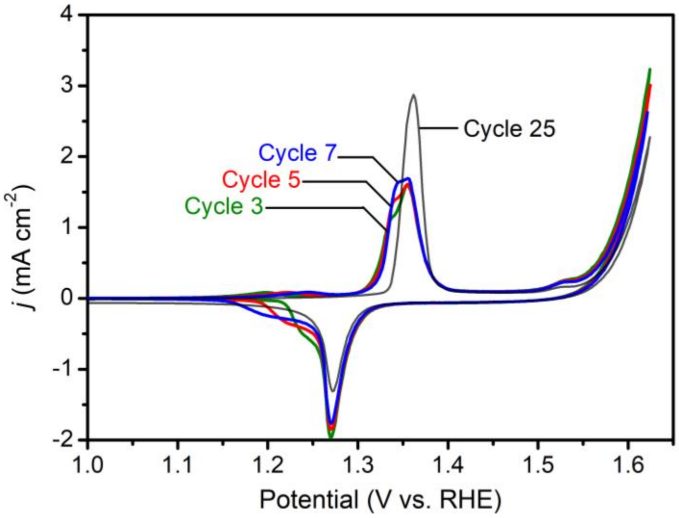

Supplementary Fig. 6 | Typical voltammetry cycles of NiO_xH_y after Co^{2+} spiking CA measurement. The movement of surface Co species into the bulk structure of NiO_xH_y affects the redox behavior of NiO_xH_y – likely due to electronic interactions between cations. In the initial cycles, Co-incorporated NiO_xH_y exhibited broadened redox peaks, which consist of two distinct oxidation and reduction peaks. This suggested an inhomogeneous local environment within the NiO_xH_y lattice. After 25 cycles, these distinct redox peaks merged into one pair of sharp peaks, which we interpret as associated with a homogeneous distribution of Co sites within the NiO_xH_y.

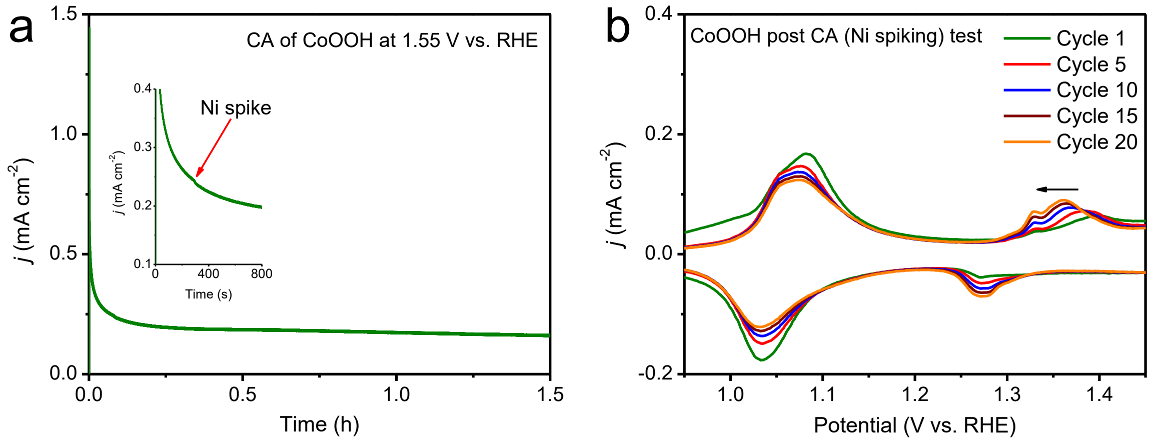

Supplementary Figure 7 | (a) Chronoamperometry (CA) measurements of CoOOH at the potential of 1.55 V vs. RHE in Ni^{2+} spiked 1 M KOH. CoOOH was first measured in purified KOH solution (inset), then $Ni(NO_3)_2$ aqueous solution was added into the electrolyte. The Ni concentration in the electrolyte was 0.1 ppm. **(b)** Voltammetry of CoO_xH_y after Ni^{2+}-spiking CA measurement in Ni^{2+} spiked 1 M KOH.

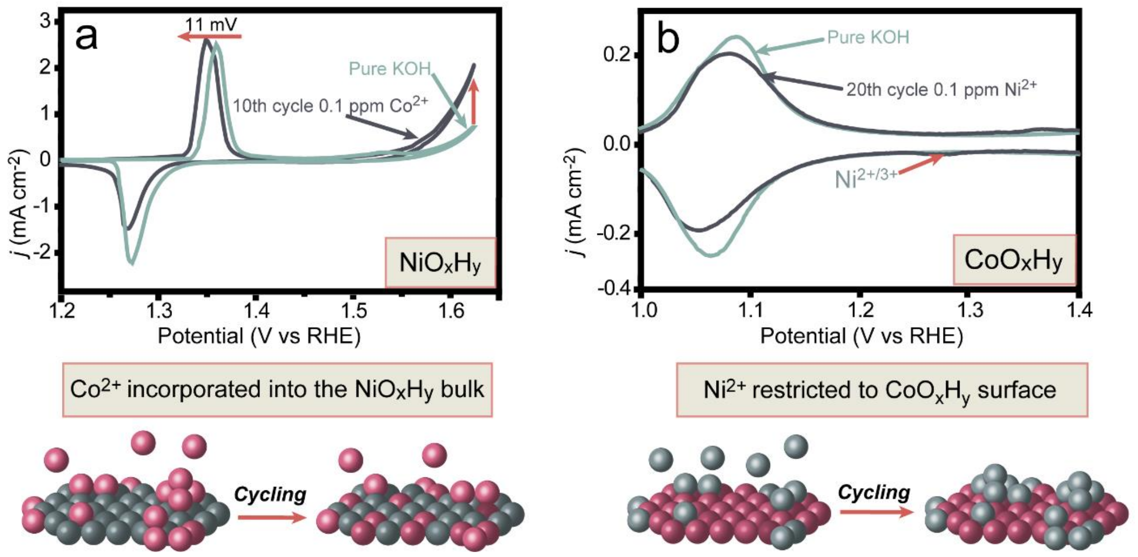

Supplementary Figure 8 | The effect of cyclic voltammetry on foreign-cation incorporation and local structure of NiO_xH_y and CoO_xH_y. (a) NiO_xH_y was first cycled at 10 mV/s in purified 1.0 M KOH, then aq. $Co(NO_3)_2$ was added to provide 0.1 ppm Co^{2+} in the electrolyte and a further 10 voltammograms were collected. **(b)** CoO_xH_y was first cycled in purified 1.0 M KOH, then aq. $Ni(NO_3)_2$ was added to provide 0.1 ppm Ni^{2+} in the electrolyte and a further 20 voltammograms were collected.

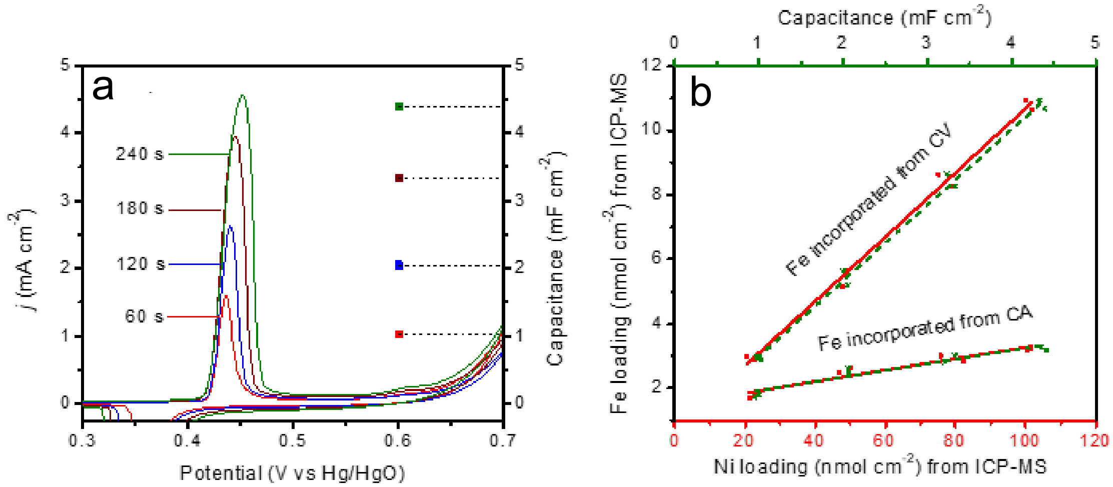

Supplementary Figure 9 | Mass loading of NiO_xH_y and incorporation of Fe. (a) Typical voltammetry of NiO_xH_y films with different mass loadings controlled by applying different electrodeposition times of 60 s (red), 120 s (blue), 180 s (wine), and 240 s (olive) under the same deposition current of -0.1 mA·cm^{-2}. The corresponding double-layer capacitance (C_{DL}) of each film was determined via AC impedance measurements at a constant potential of 0.6 V vs Hg/HgO. **(b)** Mass of Fe incorporated under CV and CA modes as a function of Ni mass loading (red symbols and solid fitted lines) and double-layer capacitance (olive symbols and dash fitted lines) of NiO_xH_y films per geometric area of substrate. The mass of Ni and Fe in the films was determined by ICP-MS. The voltammetry measurements were performed in Fe-spiked 1 M KOH for 10 cycles. The chronoamperometry measurements were performed in Fe-spiked 1 M KOH until maximum OER current was reached.

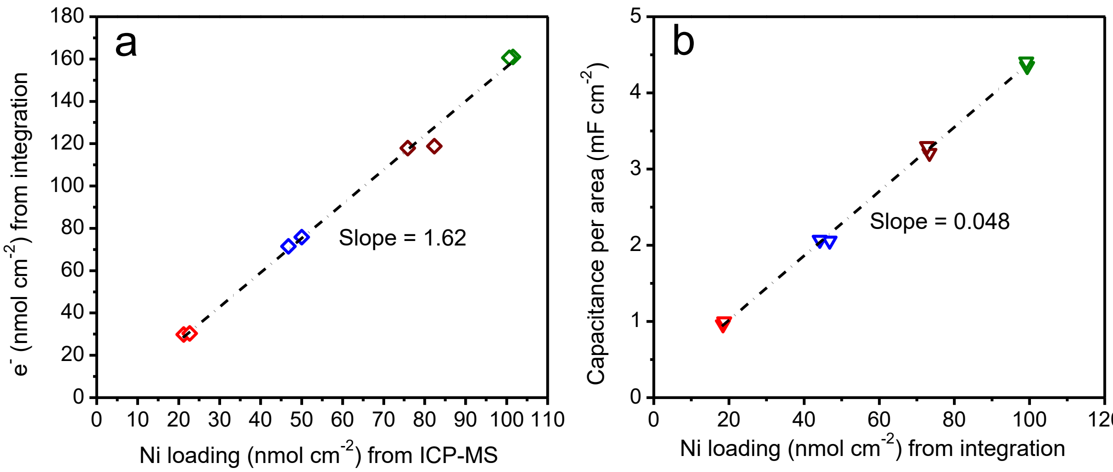

Supplementary Figure 10 | (a) Moles of electrons from integration of the anodic peak in the voltammograms as a function of the moles of Ni determined by ICP-MS analysis for NiO_xH_y films with different mass loading. The mass of NiO_xH_y films is controlled by different electrodeposition time of 60 s (red), 120 s (blue), 180s (wine), and 240 s (olive) under the same deposition current of -0.1 mA·cm^{-2}. From the linear fitting, there was ~1.6 e^- transferred per Ni during the redox process, consistent with previous findings. **(b)** The correlation between the double-layer capacitance (C_{DL}) and the mass loading for the NiO_xH_y films. The linear increase of electrons transferred and C_{DL} with the mass of films indicate that the films are electrolyte-permeable and electron-accessible throughout the mass loading range.

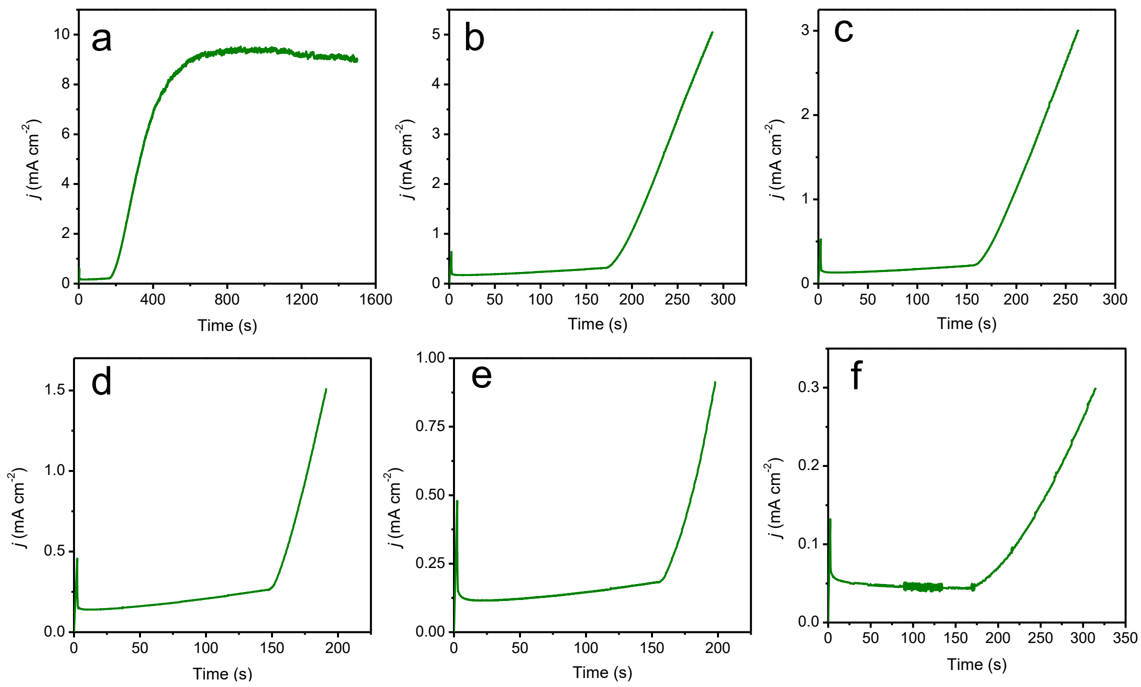

Supplementary Figure 11 | Representative chronoamperometry (CA) tests of NiOOH at an overpotential of 300 mV in Fe-spiked 1 M KOH solution. $Fe(NO_3)_3$ aqueous solution was added into KOH electrolyte at the initial stage of CA tests. The Fe concentration in the electrolyte was 0.1 ppm. CA tests were stopped after reaching current densities of **(a)** the maximum OER current density, **(b)** 5 $mA{\cdot}cm^{-2}$, **(c)** 3 $mA{\cdot}cm^{-2}$, **(d)** 1.5 $mA{\cdot}cm^{-2}$, **(e)** 0.9 $mA{\cdot}cm^{-2}$, **(f)** 0.3 $mA{\cdot}cm^{-2}$. The iR_u potential drop was corrected in real time using the manual *iR* compensation mode. Commercial semiconductor-grade KOH was used for **(a)-(e)**; purified "Fe-free" KOH solution was used for **(f)**.

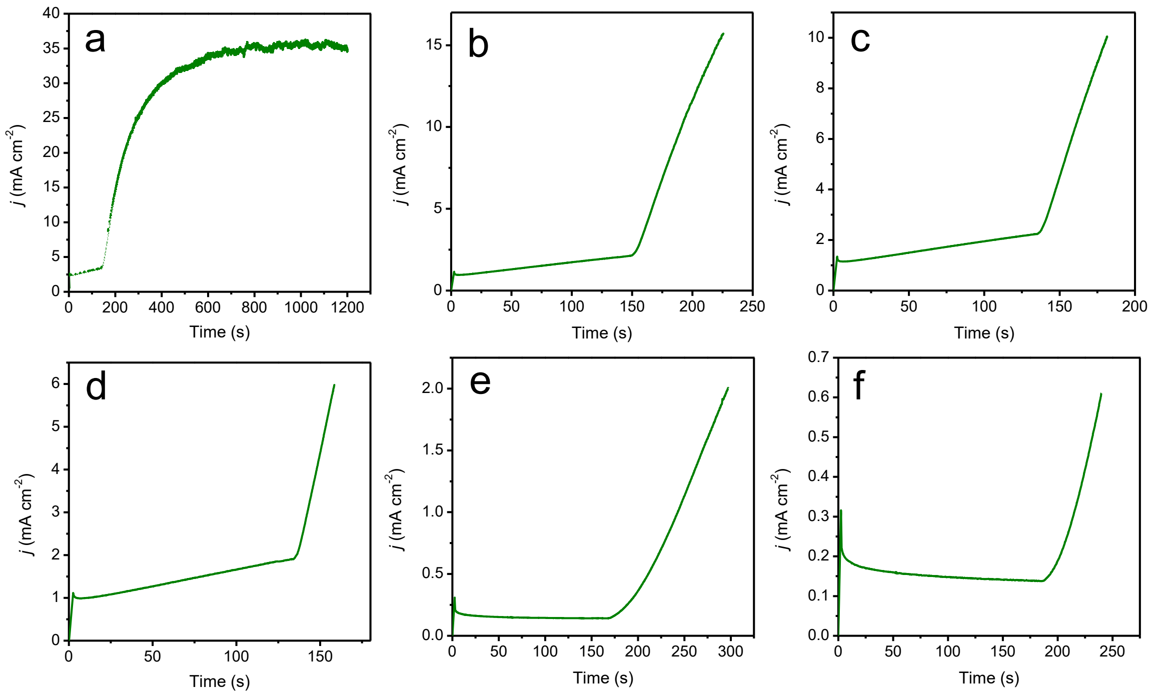

Supplementary Figure 12 | Representative chronoamperometry (CA) tests of NiOOH at an overpotential of 350 mV in Fe-spiked 1 M KOH solution. $Fe(NO_3)_3$ aqueous solution was added into KOH electrolyte at the initial stage of CA test. The Fe concentration in the electrolyte was 0.1 ppm. CA tests were stopped after reaching current densities of **(a)** the maximum OER current density, **(b)** 16 mA·cm^{-2}, **(c)** 10 mA·cm^{-2}, (d) 6 mA·cm^{-2}, **(e)** 2 mA·cm^{-2}, and **(f)** 0.6 mA·cm^{-2}. The iR_u potential drop was corrected in real time using the manual *iR* compensation mode. Commercial semiconductor grade KOH was used for **(a)-(d)**; Purified "Fe-free" KOH solution was used for **(e)-(f)**.

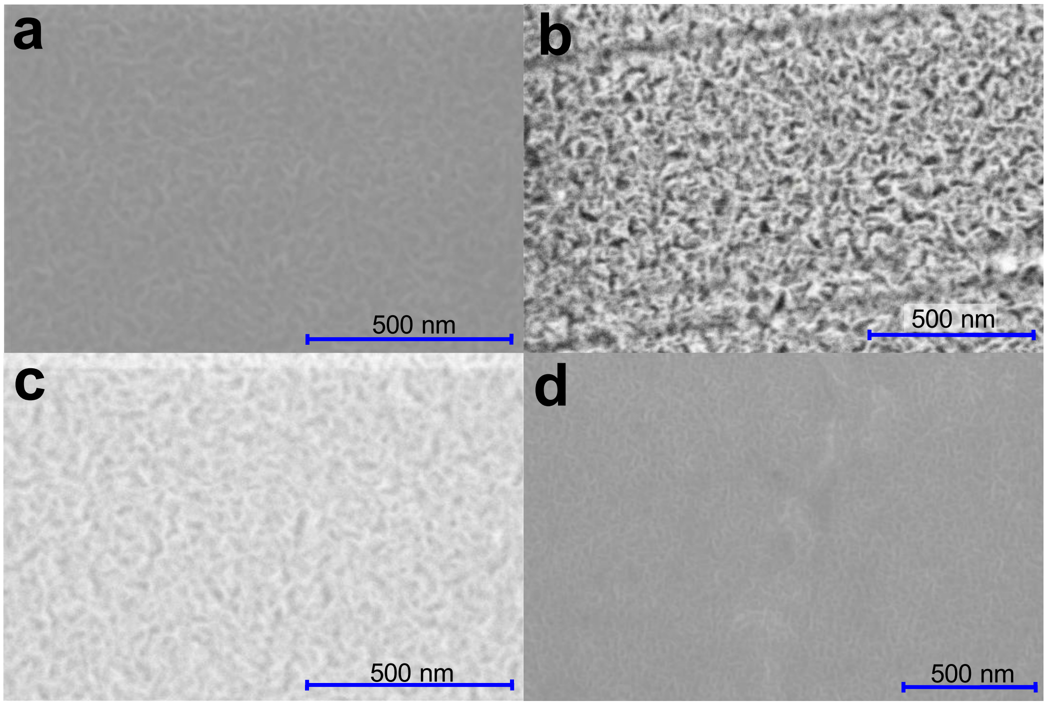

Supplementary Figure 13 | SEM images of the surface of an electrodeposited NiO_xH_y prepared as described in the Methods section. **(a, c, d)** are all the images of the same spot on a single film, but captured with different detectors. **(b)** shows a different spot on the same film, but shown with high contrast to capture the porosity of the film.

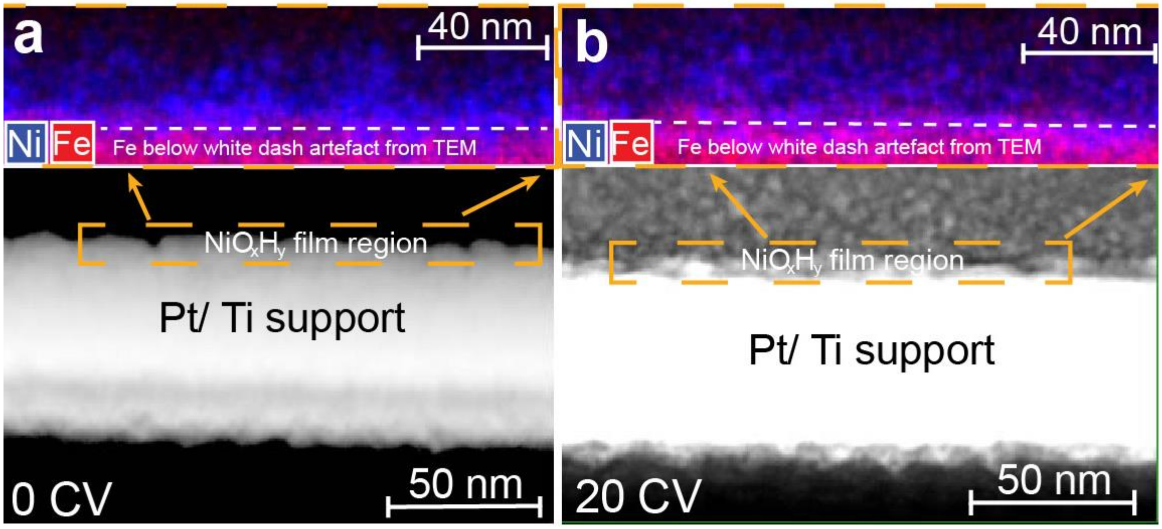

Supplementary Figure 14 | TEM/EDX of cross-sections of a NiO_xH_y film electrodeposited as described in the methods sections and with Fe incorporated by electrolyte spiking during constant potential polarization at 1.55 V vs RHE. Lamellar cross sections were cut and thinned with a Ga focused ion beam from films of morphology like those shown in SI figure 12. **(a)** shows a film without further cycling after Fe incorporation and **(b)** shows a separate but analogous film with 20 cycles after Fe incorporation. We note that the high Fe concentration toward the bottom of the EDX picture is partially due to system peaks arising from Fe in the lenses of the TEM instrument. This signal scales with the scattering intensity of nearby high mass elements.

Because substrate is platinum, Fe appears more intensely in the areas closest to the substrate. Regions below the dashed boxes are all either the Pt substrate, Ti adhesion layer, or the glass slide these are coated on, so their EDX profiles are omitted.

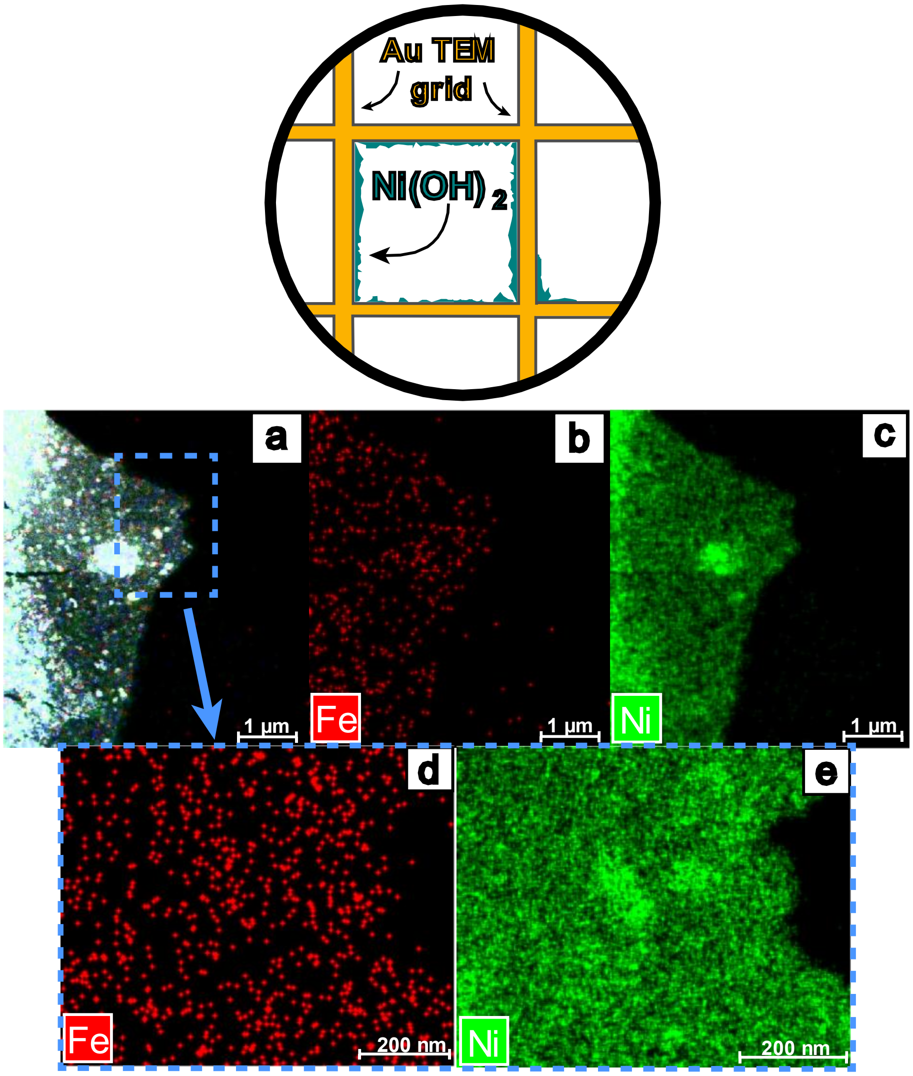

Supplementary Figure 15 | TEM-EDX images of NiO_xH_y electrodeposited directly onto a gold TEM grid. Fe was then adsorbed during chronoamperometry at 1.55 V vs RHE. The topmost image illustrates qualitatively how $Ni(OH)_2$ flakes (teal) grow directly onto the TEM grid (yellow) and span into the interior grid space. A single flake was selected and used to generate images **(a-e)** which are representative of other locations sampled on the deposited flakes. Fe is relatively sparsely distributed relative to Ni and appears as separated clusters; however, we cannot discriminate between specific bulk surface position of the iron from these images. Further, the low concentrations of Fe make it difficult to say much definitively about the structure of Fe.

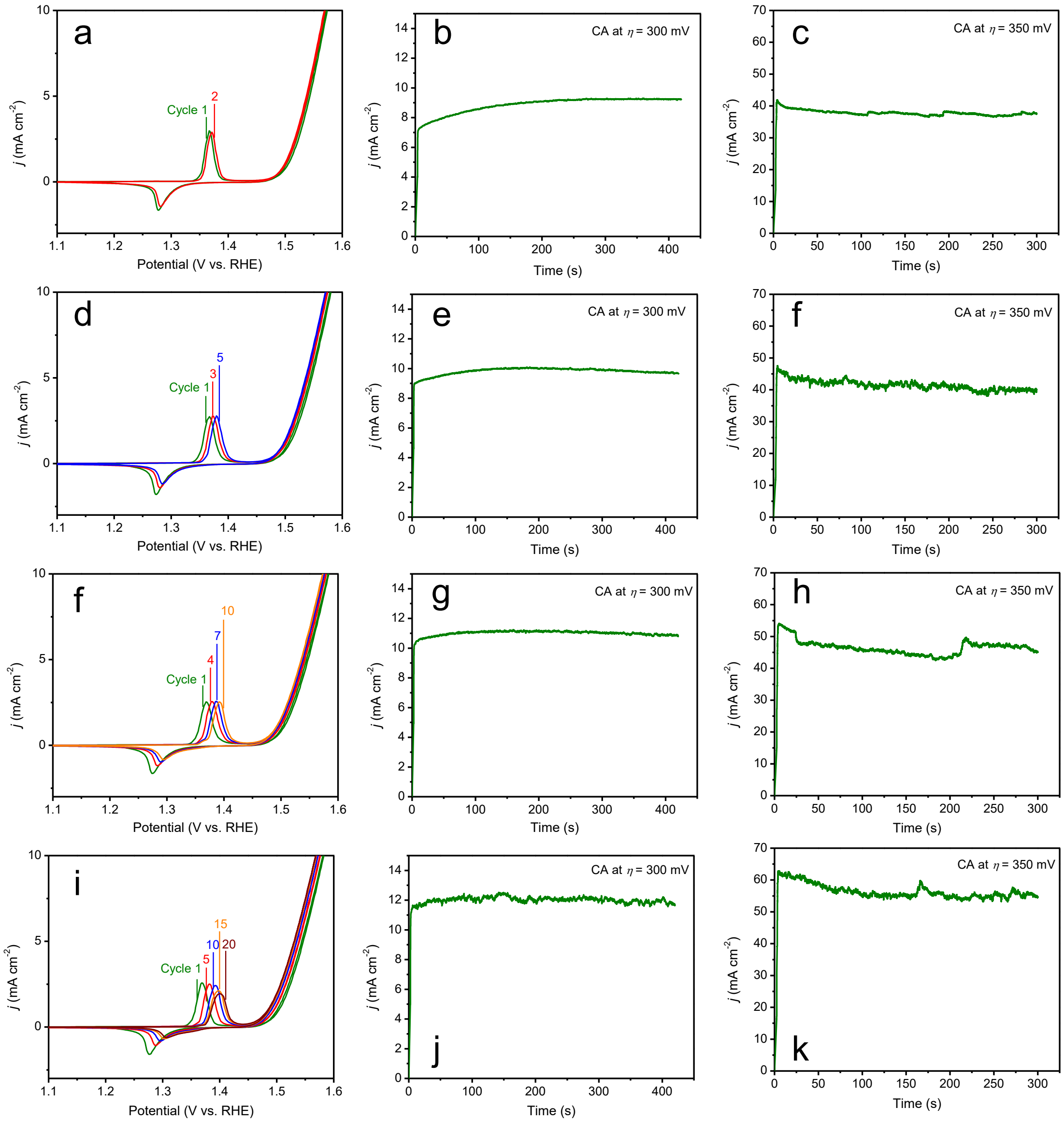

Supplementary Figure 16 | To calculate the TOF_{Fe} of mixed (bulk and surface) Fe species, Ni(Fe)O_xH_y films obtained from initial CA-spiking route were further cycled for **(a)** 2, **(d)** 5, **(f)** 10, and **(i)** 20 cycles. Then CA measurements at overpotentials of **(b)**, **(e)**, **(g)**, **(f)** 300 mV, and **(c)**, **(f)**, **(h)**, **(k)** 350 mV were used to collect the OER currents.

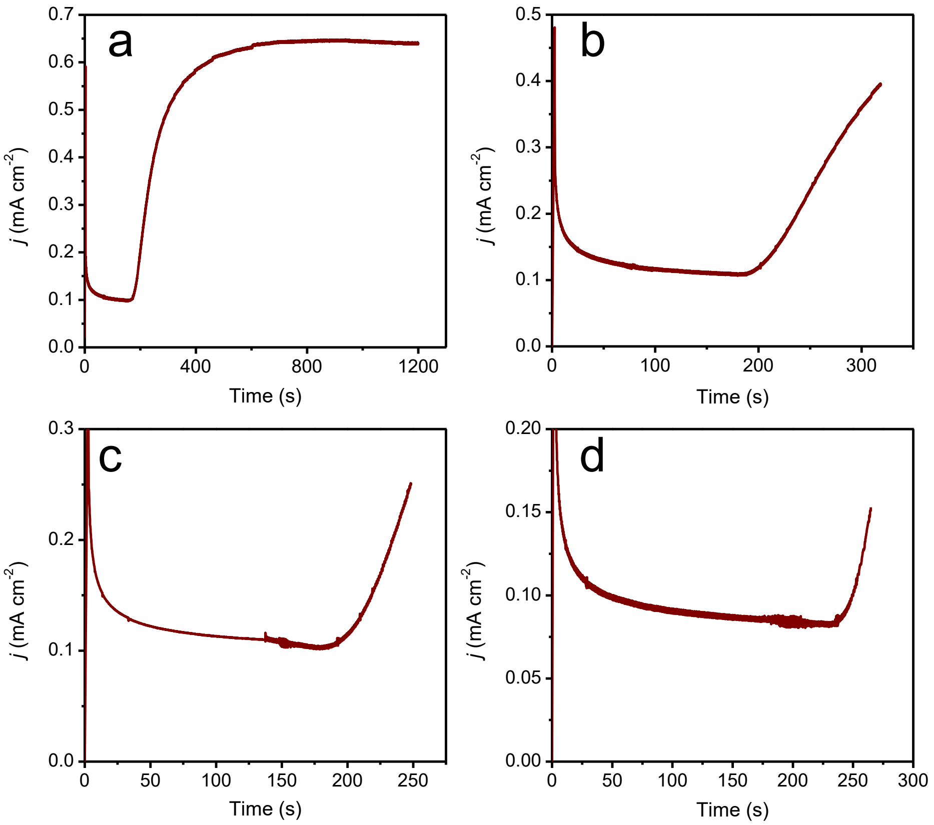

Supplementary Figure 17 | Representative chronoamperometry (CA) tests of CoOOH at an overpotential of 300 mV in Fe-spiked 1 M KOH solution. $Fe(NO_3)_3$ aqueous solution was added into KOH electrolyte at the initial stage of CA tests. The Fe concentration in the electrolyte was 0.1 ppm. CA tests were stopped after reaching current densities of **(a)** the maximum OER current density, **(b)** 0.4 mA·cm^{-2}, **(c)** 0.25 mA·cm^{-2}, **(d)** 0.15 mA·cm^{-2}. The iR_u potential drop was corrected in real time using the manual *iR* compensation mode. Commercial semiconductor grade) KOH was used for **(a)-(c)**; purified "Fe-free" KOH solution was used for **(d).**

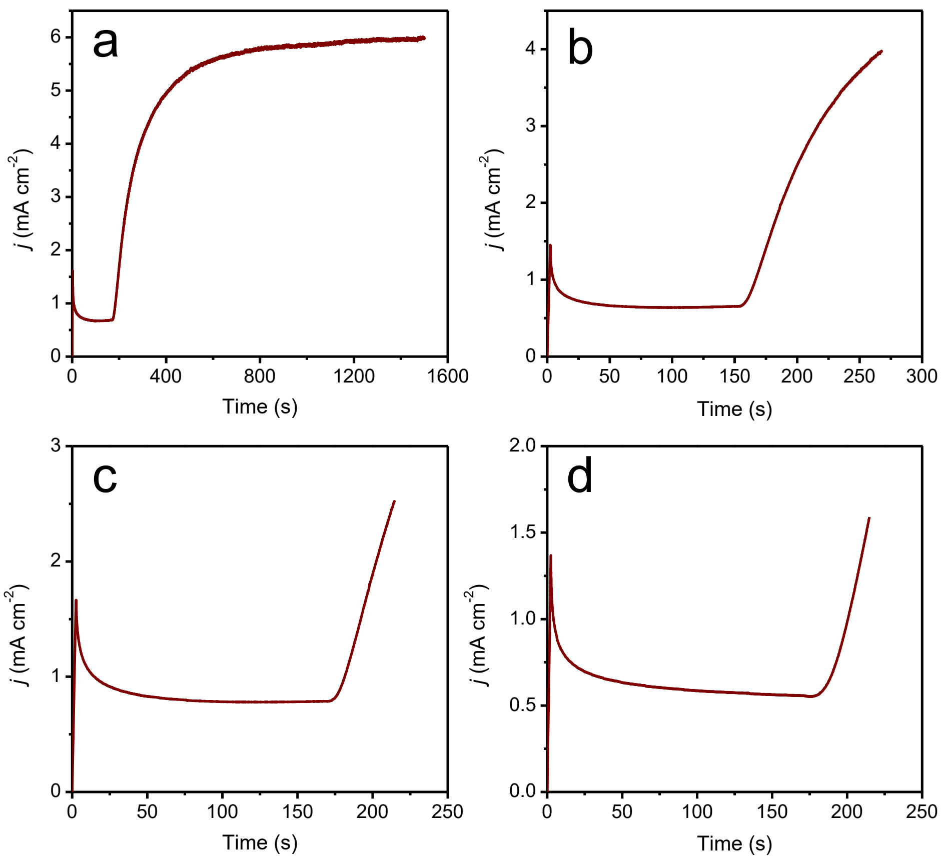

Supplementary Figure 18 | Representative chronoamperometry (CA) tests of CoOOH at an overpotential of 350 mV in Fe-spiked 1 M KOH solution. $Fe(NO_3)_3$ aqueous solution was added into KOH electrolyte at the initial stage of CA tests. The Fe concentration in the electrolyte was 0.1 ppm. CA tests were stopped after reaching current densities of **(a)** the maximum OER current density, **(b)** 4 mA·cm^{-2}, **(c)** 2.5 mA·cm^{-2}, **(d)** 1.5 mA·cm^{-2}. The iR_u potential drop was corrected in real time using the manual *iR* compensation mode. Commercial semiconductor grade) KOH was used for **(a)-(c)**; purified "Fe-free" KOH solution was used for **(d).**

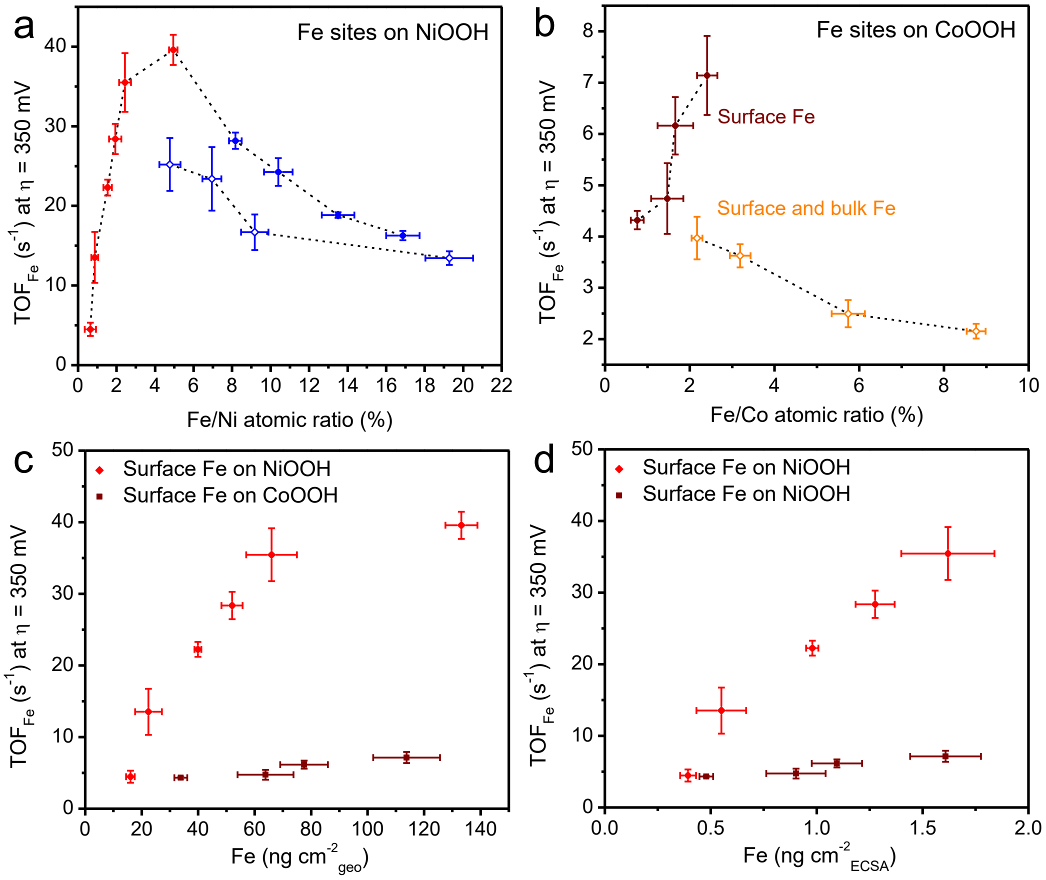

Supplementary Figure 19 | The intrinsic activity comparison of Fe sites reflected by the turnover frequency (TOF_{Fe}) at $\eta = 350$ mV (complementing the $\eta = 300$ mV data in the main text). The TOF_{Fe} is calculated based on the mass of all Fe sites determined by ICP-MS analysis. **(a)** Correlation between the TOF_{Fe} and Fe/Ni atomic ratio. The closed red symbols are for surface Fe sites obtained from CA-Fe spiking method, and the closed blue symbols stand for mixed (surface and bulk) Fe sites obtained from further voltammetry cycling. The open blue symbols represent co-deposited $Ni_{1-x}Fe_xOOH$. **(b)** Correlation between the TOF_{Fe} and Fe/Co atomic ratio. The closed wine symbols stand for surface Fe sites obtained from CA-Fe spiking method and the open orange symbols stand for Fe sites in co-deposited $Co_{1-x}Fe_xOOH$. **(c)** The correlation between the TOF_{Fe} of surface Fe sites on NiOOH (red symbols) and CoOOH (wine symbols) and the adsorbed Fe mass loading normalized by the geometric area of substrate. **(d)** The correlation between the TOF_{Fe} of surface Fe sites on NiOOH (red symbols) and CoOOH (wine symbols) and the adsorbed Fe mass loading normalized by the electrochemically active surface areas of NiOOH and CoOOH films. Error bars are standard deviations based on samples measured in triplicate.

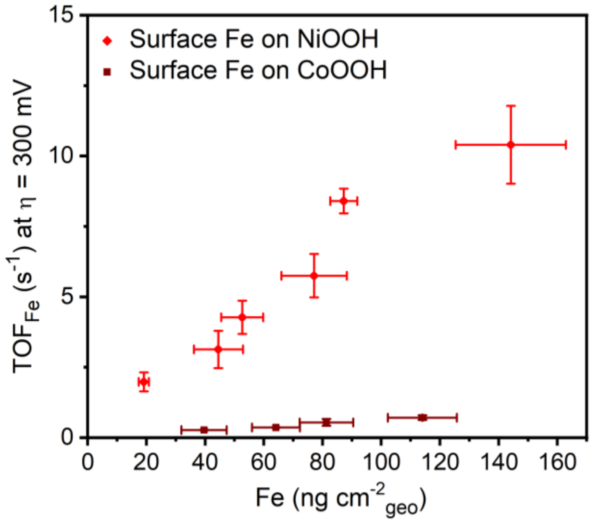

Supplementary Figure 20 | The correlation between the TOF_{Fe} (η = 300 mV) of surface Fe sites on NiOOH (red symbols) and CoOOH (wine symbols) and the adsorbed Fe mass loading normalized by the geometric area of substrate. Error bars are one standard deviation from the average of triplicate measurements.

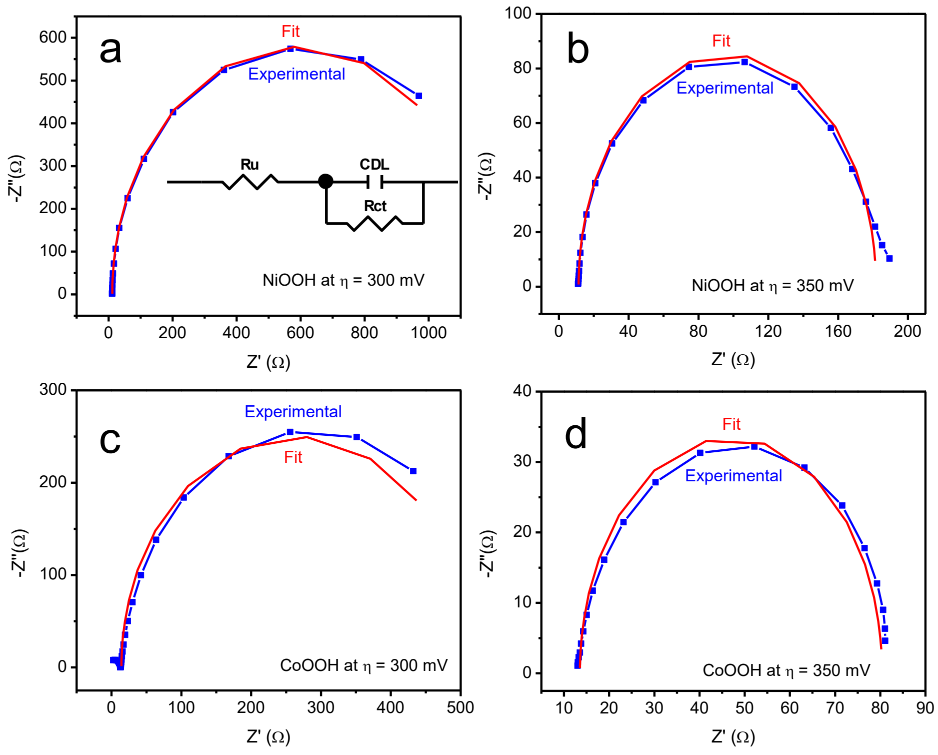

Supplementary Figure 21 | Potentioelectrochemical impedance spectroscopy (PEIS) measurements used to determine the double-layer capacitance of NiOOH film at the overpotential of **(a)** 300 mV and **(b)** 350 mV, and the CoOOH films at the overpotential of **(c)** 300 mV and **(d)** 350 mV. The equivalent circuit shown in the inset of **(a)** was used to fit the experimental impedance results. Least-squares data fitting was conducted to determine the circuit elements (EC Lab, Biologic).

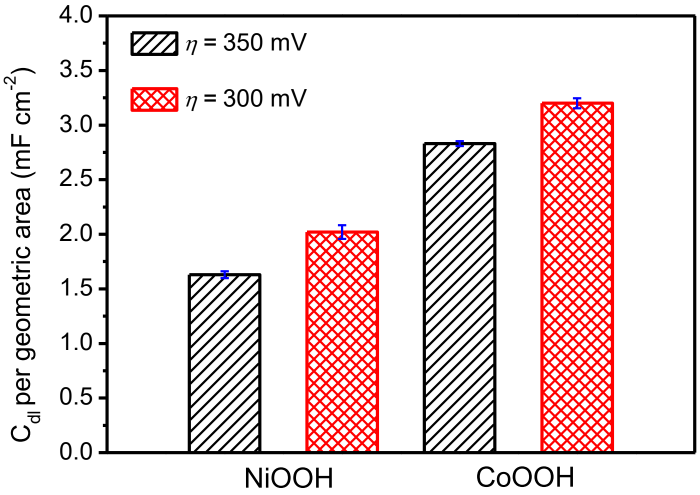

Supplementary Figure 22 | Double-layer capacitance (C_{dl}) per geometric area from PEIS equivalent circuit fitting for NiOOH and CoOOH films at the overpotentials of 300 mV and 350 mV. Error bars are one standard deviation from the average of triplicate measurements.

Supplementary Note 1: X-ray absorption measurement discussion

A NiOOH film was electrodeposited on a polycrystalline Pt plate at -0.1 mA cm^{-2} for 120 s from a 0.1 M $Ni(NO_3)_3 \cdot 6H_2O$ solution. Then 0.1 M KOH was filled into the cell. $Fe(NO_3)_3$ was added into the flowing electrolyte reservoir to a final concentration of ~150 ppb. The electrode potential was then immediately stepped and held at 0.68 V vs Hg/HgO and XAS spectra were simultaneously recorded (Fe@0.68V, after 0 CVs). Spectra were next obtained at 0.60 V vs. Hg/HgO (Fe@0.60 V, after 0 CVs). Following a short open-circuit potential step, the sample was cycled 17 times to introduce Fe into the internal sites of the NiO_xH_y structure, and finally held at 0.68 V vs. Hg/HgO (Fe@0.68V, after 17 CVs) for a final set of XAS spectra. We focus on the analysis of the Fe *K*-edge of the film at 0.68 V vs. Hg/HgO before and after cycling. There were no significant changes in the edge position of shape of the XANES (Fig. S24).

The lack of significant changes to the Fe spectra show that in all cases the nominal valence state of the Fe is similar and comparison to hematite (α-Fe_2O_3) indicates a valence of 3+. This is consistent with picture of structural differences between the FeOx surface cluster active sites and internal sites driving activity differences.

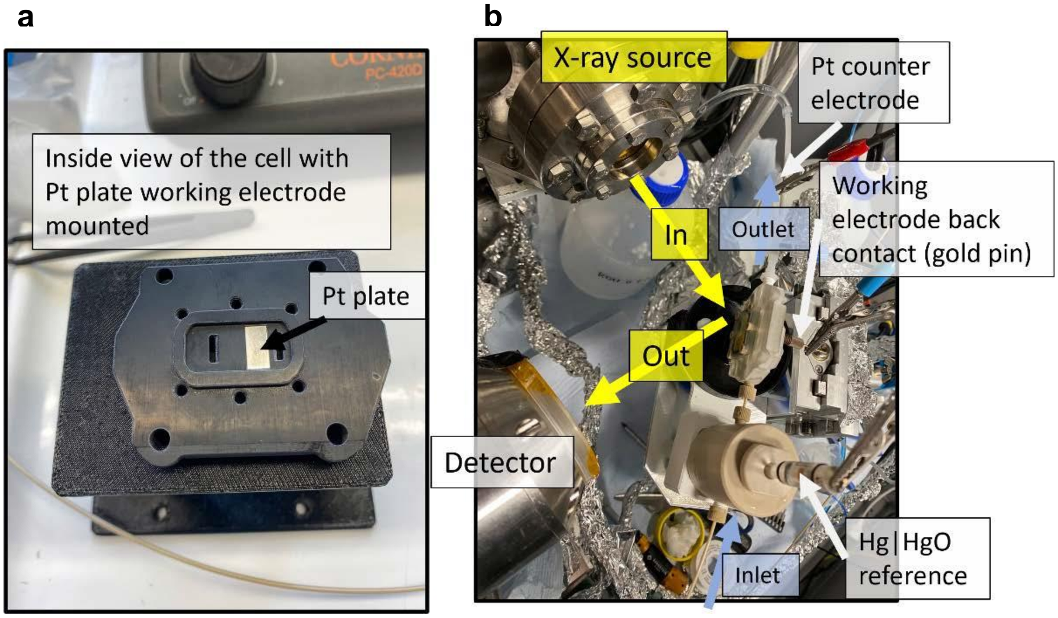

Supplementary Figure 23 | (a) inside view of the *in-situ* XAS flow cell with Pt plate working electrode mounted. Electrical contact was made from the back (underneath the plate in left image) using a gold pin. $Ni(OH)_2$ was deposited directly onto the Pt plate in a separate conventional cell according to the method presented in the main text prior to mounting into the flow cell. **(b)** Complete experimental set-up with flow cell connected to flowing electrolyte and counter and reference electrodes.

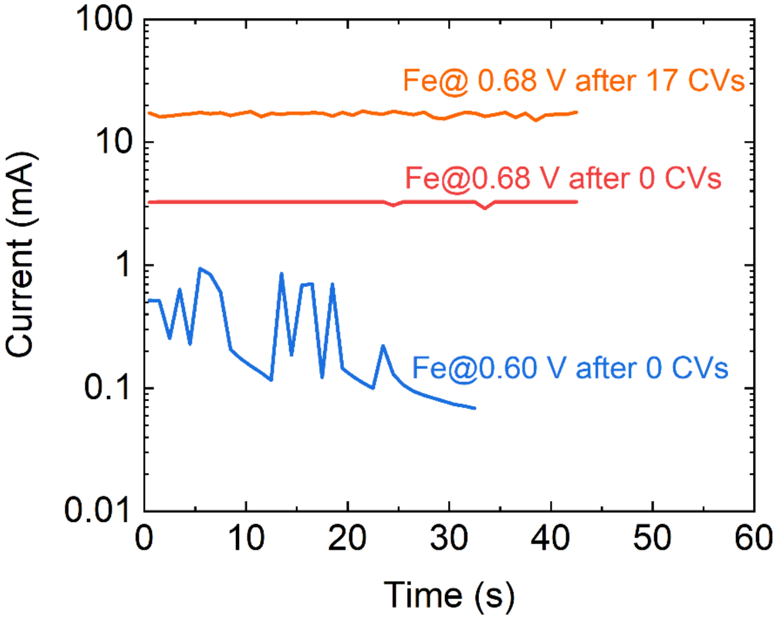

Supplementary Figure 24 | Constant potential data acquired in the *operando* XAS cell immediately after Fe spiking (red – 0.68 V; blue – 0.60 V) and after 17 CVs (orange).

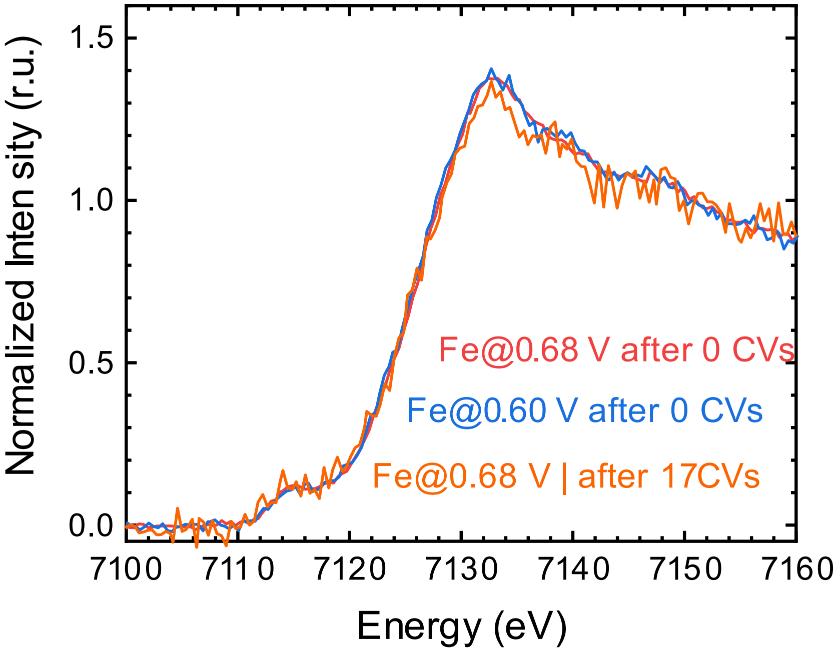

Supplementary Figure 25 | XANES at the Fe-K edge of NiO_xH_y electrodeposited on a Pt plate with Fe incorporated by spiking ~170 ppb Fe into the electrolyte reservoir during a short (< 2 min) period at open circuit. Fe spiking was done during open circuit because of concerns that the *operando* cell would dry out and over-polarize during CA, thereby destroying the film. After spiking the reservoir, the sample was immediately polarized to 0.68 V vs. Hg/HgO and an XAS spectra was obtained (red) followed by another set of spectra obtained at 0.60 V vs Hg/HgO (blue) with a short (< 90 s) OCV period between this and the 0.68 V spectra. 17 cycles were performed after the 0.60 V scans and another spectrum was obtained at 0.68 V vs Hg/HgO (orange). The lack of significant changes to the Fe spectra shows that in all cases the nominal valence state of 3+ of the Fe is unchanged, consistent with our picture of structural differences between the FeO_x surface cluster active sites and internal sites driving activity differences.

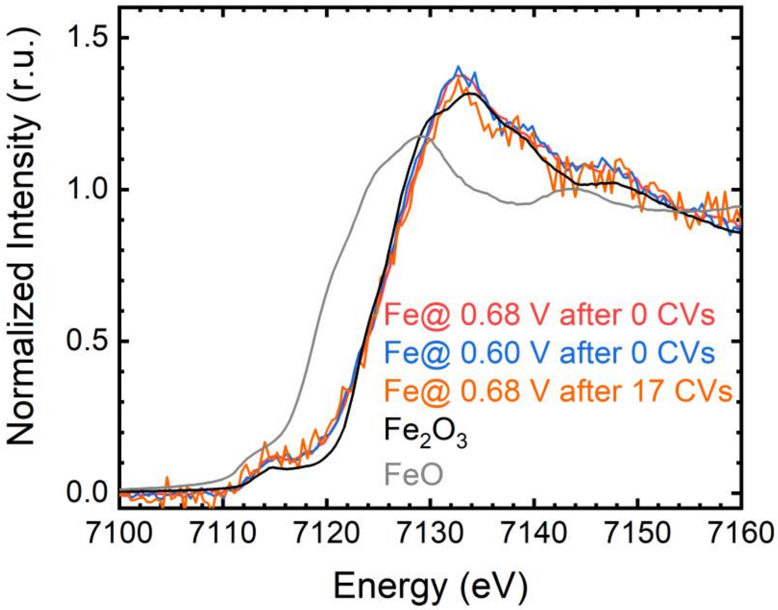

Supplementary Figure 26 | Comparison of experimental data with reference materials Fe_2O_3 and FeO. The similarity in shape and position of the data with Fe_2O_3 confirms that the valence of Fe species incorporated by spiking during CA is 3+.

Supplementary Table 1. Parameters of the data extraction and fits.

Fit ID	Sample	EXAFS range (eV)	# knots	Fit range (Å^{-1})	Degree of freedom
CA068-B	Fe@0.68, after 0 CVs	10 – 680	10	3.6 – 7.8	7
CA068-A	Fe@0.68, after 0 CVs	10 – 680	10	3.2 – 7.8	9
CA060-A	Fe@0.60, after 0 CVs	15 - 660	10	3.2 – 7.8	7
CV068-A	Fe@0.68, after 17 CVs	20 – 690	7	3.2 – 7.8	7

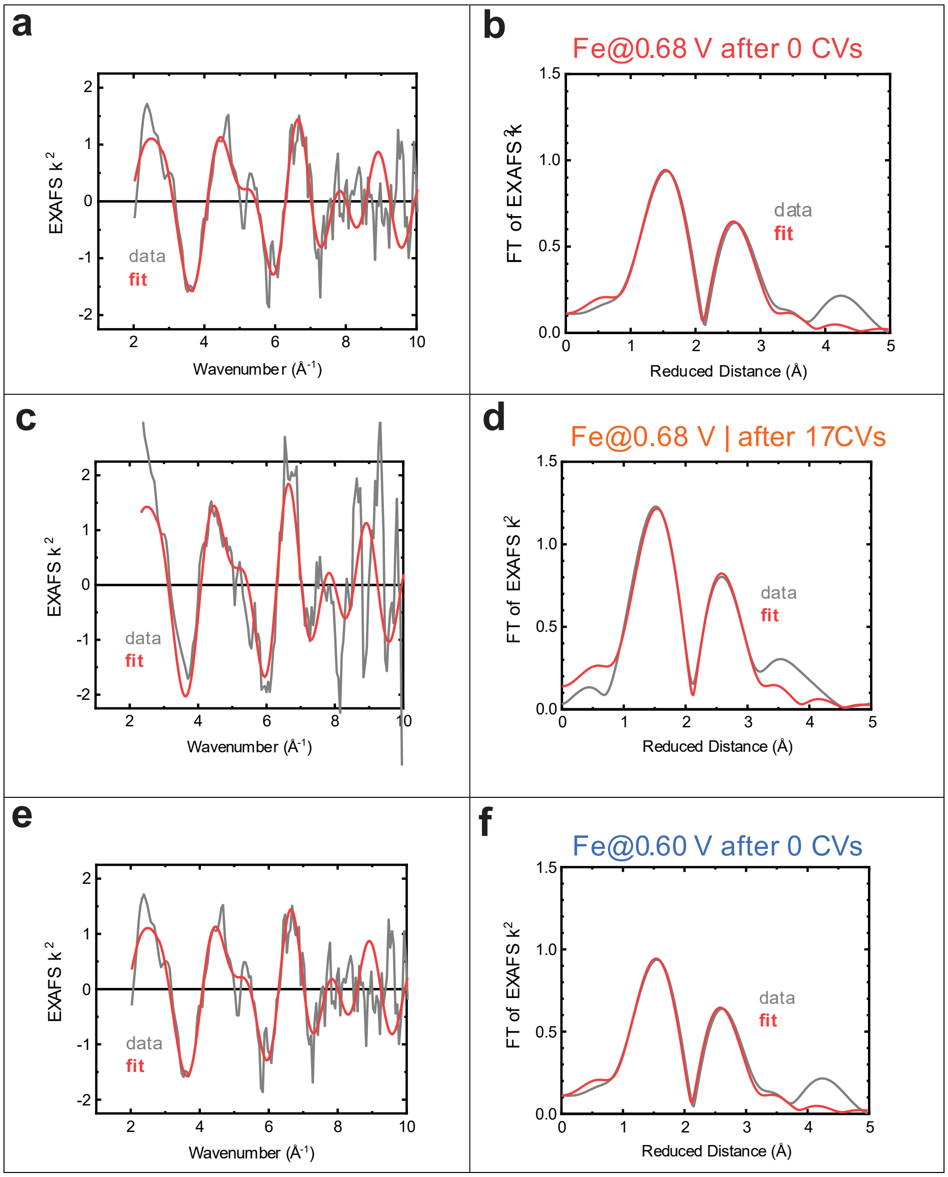

Supplementary Figure 27 | Fits in *k*-space and real space of **(a,b)** Fe@0.68 V vs Hg/HgO after 0 cycles – the red curve in SI figure 22 **(c,d)** Fe@0.68 V vs Hg/HgO after 17 cycles, the orange curve in SI figure 22 and **(e,f)** Fe@0.60 V vs Hg/HgO after 0 cycles, the blue curve in SI figure 22. Note that the actual fits were always performed in *k*-space and then Fourier transformed between 22 to 244 eV (2.4 – 8.0 $Å^{-1}$) using a cosine window on the first and last 10% of the data. All data above was transformed identically.

Supplementary Table 2. EXAFS fit results

		Fe-O*			Fe-M**		
Exp. ID	Sample History	N	R (Å)	Rf' (%)	N	R (Å)	Rf' (%)
CA068-A	Spectra collected on NiOOH (sample A) held at 0.68 V vs. Hg/HgO with 150 ppb Fe3+ in the electrode	4.26 ± 0.2	1.953 ± 0.004	0.82	5.08 ± 0.3	2.988 ± 0.005	0.82
CA068-B	Same as CA068-A except analysed with different fit range, notice similar fit results	4.09 ± 0.2	1.951 ± 0.005	1.31	5.10 ± 0.3	2.987 ± 0.006	1.31
CA060-A	Sample A was then allowed to rest for 90 s at OCV, and another spectra collected at 0.60 V vs Hg/HgO	4.29 ± 0.1	1.941 ± 0.003	0.49	5.03 ± 0.2	2.975 ± 0.004	0.49
CV068-A	Sample A was then subjected to 17 voltammogram cycles in the same electrolyte with 150 ppb Fe3+	5.56 ± 0.6	1.939 ± 0.011	5.67	6.38 ± 1.1	2.974 ± 0.014	5.67

Fixed parameters: ΔE0 = 4 eV; S02 = 0.65; * 2σ2 = 0.05 Å2. ** 2σ2 = 0.01 Å2. Where ΔE0 is the energy for aligning XAS data to an absolute energy grid for fitting, S02 is the amplitude reduction factor, and σ2 is the Debye-Waller factor. Note that EXAFS cannot distinguish Fe (atomic number 24) from Ni (atomic number 26). The information whether Fe is in a NiO_x host or FeO_x host is derived from the bond length, which can be determined more precisely than coordination numbers by EXAFS analysis.

XAS interpretation. The EXAFS was extracted and fit as detailed in the experimental section with parameters in Table 1. The restriction to 7.8 $Å^{-1}$ in the fits was necessary due to noise and/or distortions in some of the samples, in particular for Fe-CV, 0.68 V. Extending the fit range on the lower end yields identical parameters within error (Table 2). The Fe-O bond length at 0.68 V vs. Hg/HgO contracted after cycling, which is consistent with the movement from (primarily) absorbed FeO_x cluster species, to a mixed $NiFeO_xH_y$ where the Fe-O bond is compressed due to integration the $NiFeO_xH_y$ mixed cation phase as discussed in the main text. However, the differences in bond length are within error and the contraction was also found after the open circuit measurement and holding the sample at 0.60 V in some cases. Further work repeating these measurements on the $NiFeO_xH_y$ and the $CoFeO_xH_y$ system with higher signal-to-noise ratio should be done to confirm the statistical significance of the bond contraction upon cycling.

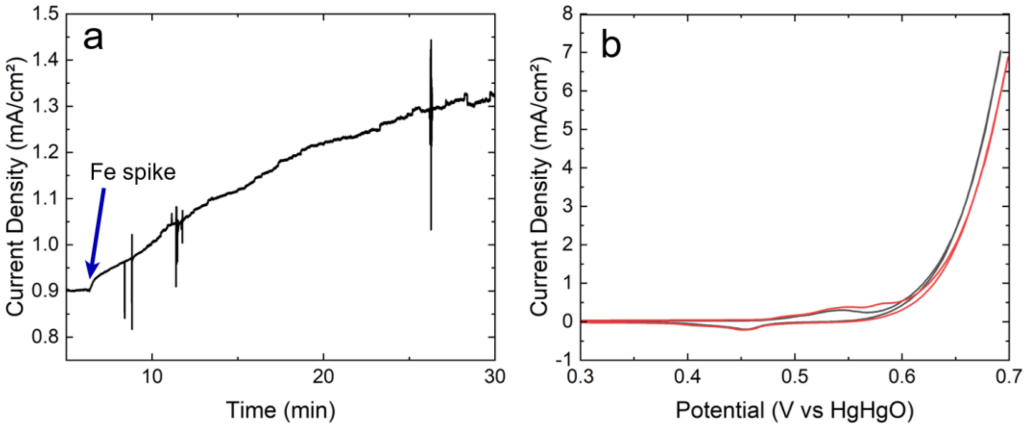

Supplementary Figure 28 | (a) Current density of a $Ni_{0.8}Fe_{0.2}O_xH_y$ film after addition of 100 ppb Fe while polarizing at 1.55 V vs RHE as was done with Fe-free films. (**b**) Cyclic voltammetry of the film immediately after deposition and before the Fe spike (red) and after the Fe-spike (black).

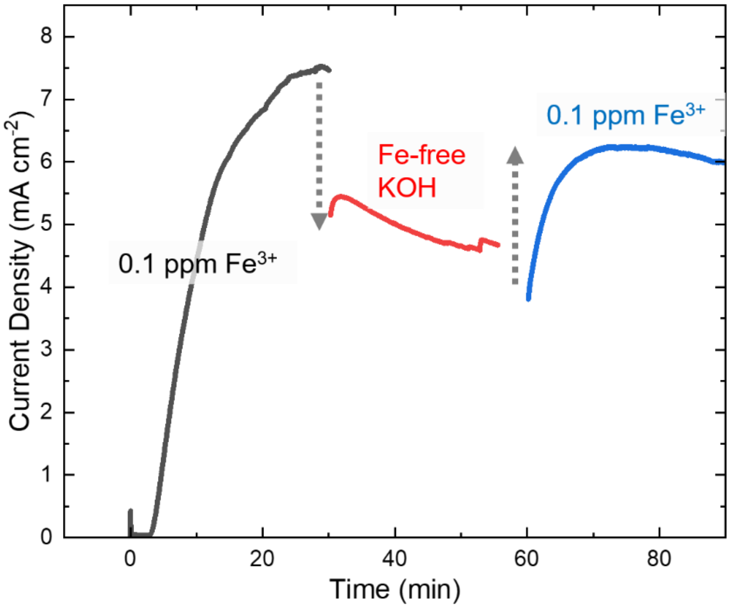

Supplementary Figure 29 | The OER current density increase at 1.55 V vs RHE of a NiO_xH_y film from addition of $Fe(NO_3)_3$ until the electrolyte concentration was nominally 0.1 ppm (black trace). This current density decreases upon placement of the electrode into Fe-free KOH (red trace) and is largely recovered (blue trace) when re-immersed in the original Fe spiked solution.

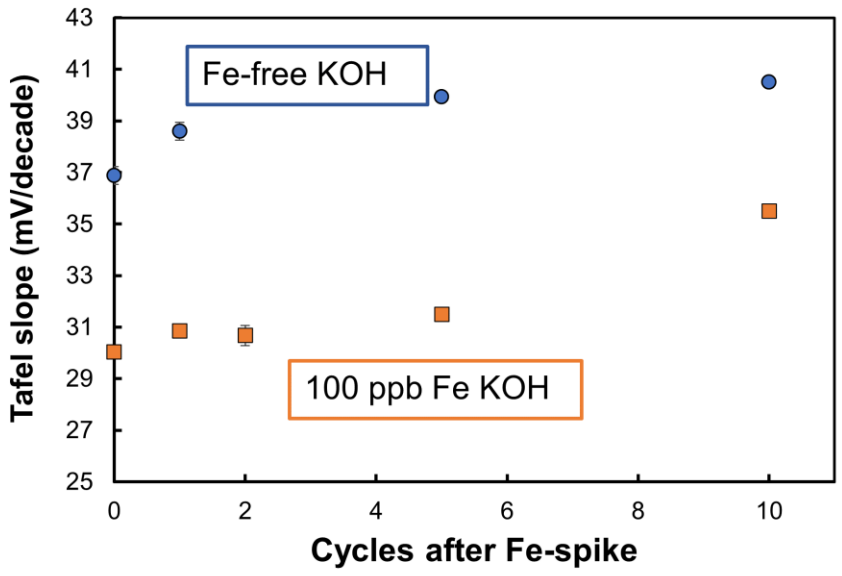

Supplementary Figure 30. Tafel slopes of CA Fe-spiked NiOOH. Data in blue was acquired in fresh Fe-free 1 M KOH after the CA Fe-spike while that in orange was kept in the electrolyte with 100 ppb Fe. Error bars are one standard deviation in the average linear fit to the data and are in some cases smaller than the data point and in general were < 1 mV decade^{-1}.

Supplementary Table 3. The ΔG and theoretical reaction overpotential (η_{th}) for various OER mechanisms given in eV or V.*

Gibbs free energy change	(0$\bar{1}$5) surface										(001) surface	
	isolated Fe-O		Fe-O-M dimer								Fe-O monomer	Fe-O-Fe dimer
	axial		axial		insertion		bridge		equatorial		axial	axial
	Fe	Ni	Fe	Ni	Fe	Ni	Fe	Ni	Fe	Ni	Fe	Fe
ΔG_1	1.60	1.62	1.73	1.80	1.73	1.80	1.45	1.41	1.16	1.48	1.84	2.01
ΔG_2	2.03	2.03	1.67	2.12	0.93	1.01	1.56	1.78	1.70	1.92	2.56	1.88
ΔG_3	-1.16	1.03	1.23	0.65	1.59	1.53	1.05	0.96	0.52	0.27	-0.14	0.37
ΔG_4	2.19	-0.03	0.22	0.36	0.67	0.58	0.86	0.76	1.47	1.46	0.30	0.47
ΔG_5	0.26	0.27	0.07	0.00	–	–	–	–	0.07	-0.20	0.36	0.18
η_{th}	0.96	0.80	0.50	0.89	0.50	0.57	0.33	0.55	0.47	0.68	1.33	0.78

*η_{th} is the theoretical overpotential of the entire reaction, which is defined as the potential needed for all the reactions steps to have negative free energies. "–" indicates that ΔG is absent for that pathway due to different number of intermediates for each mechanism. Columns with "Fe" mean that the dimer has two Fe atoms and columns with "Ni" mean that the dimer has one Fe atom and one Ni atom.

The elementary steps of the above mechanisms are represented as follows:

Axial Mechanism and Equatorial Mechanism

Step 1: $\mathbf{M_{sur\_H_2O} \longrightarrow M_{sur\_OH} + (H^+ + e^-)}$

Step 2: $\mathbf{M_{sur\_OH} \longrightarrow M_{sur\_O} + (H^+ + e^-)}$

Step 3: $\mathbf{M_{sur\_O} + H_2O \longrightarrow M_{sur\_OOH} + (H^+ + e^-)}$

Step 4: $\mathbf{M_{sur\_OOH} \longrightarrow M_{sur\_OO} + (H^+ + e^-)}$

Step 5: $\mathbf{M_{sur\_OO} + H_2O \longrightarrow M_{sur\_H_2O} + O_2}$

Bridge Mechanism

Step 1: $\mathbf{M_{sur\_OH} \longrightarrow M_{sur\_O} + (H^+ + e^-)}$

Step 2: $\mathbf{M_{sur\_O} + H_2O \longrightarrow M_{sur\_OOH} + (H^+ + e^-)}$

Step 3: $\mathbf{M_{sur\_OOH} \longrightarrow M_{sur\_OO} + (H^+ + e^-)}$

Step 4: $\mathbf{M_{sur\_OO} + H_2O \longrightarrow M_{sur\_OH} + O_2 + (H^+ + e^-)}$

Insertion Mechanism

Step 1: $\mathbf{M_{sur\_H_2O} \longrightarrow M_{sur\_OH} + (H^+ + e^-)}$

Step 2: $\mathbf{M_{sur\_OH} + H_2O \longrightarrow M_{sur\_H_2O\_O_p} + (H^+ + e^-)}$

Step 3: $\mathbf{M_{sur\_H_2O\_O_p} \longrightarrow M_{sur\_OH\_O_p} + (H^+ + e^-)}$

Step 4: $\mathbf{M_{sur\_OH\_O_p} + H_2O \longrightarrow M_{sur\_H_2O} + O_2 + (H^+ + e^-)}$

$\mathbf{M_{sur}}$ represents the metal site on the surface where the reaction is taking place

$\mathbf{M_{sur\_\mathit{X}}}$ (x=H_2O, OH, O, OOH, OO) represents the reactive metal site with respective intermediate.

$\mathbf{M_{sur_X_O_p}}$represents the reactive metal site with oxygen penetrated at the bridge position along with the intermediate at the metal site.

Reaction free energies are calculated using the formulae:

$$\Delta G_1 = E^*_{OH} - E^*_{H_2O} + \frac{1}{2}E_{H_2} + \frac{1}{2}(ZPE - T.S)_{H_2}$$

$$\Delta G_2 = E^*_{O} - E^*_{OH} + \frac{1}{2}E_{H_2} + \frac{1}{2}(ZPE - T.S)_{H_2}$$

$$\Delta G_3 = E^*_{OOH} - E^*_{O} + \frac{1}{2}E_{H_2} - E_{H_2O} + \frac{1}{2}(ZPE - T.S)_{H_2} - (ZPE - T.S)_{H_2O}$$

$$\Delta G_4 = E^*_{OO} - E^*_{OOH} + \frac{1}{2}E_{H_2} + \frac{1}{2}(ZPE - T.S)_{H_2}$$

$$\Delta G_5 = E^*_{H_2O} - E^*_{OO} + E_{O_2} - E_{H_2O} + (ZPE - T.S)_{O_2} - (ZPE - T.S)_{H_2O}$$

Where E^*_X is the VASP energy of the intermediates that includes the ZPE corrections, E_x is the energy of the gaseous molecules, ZPE and TS are the respective zero point and entropy correction.

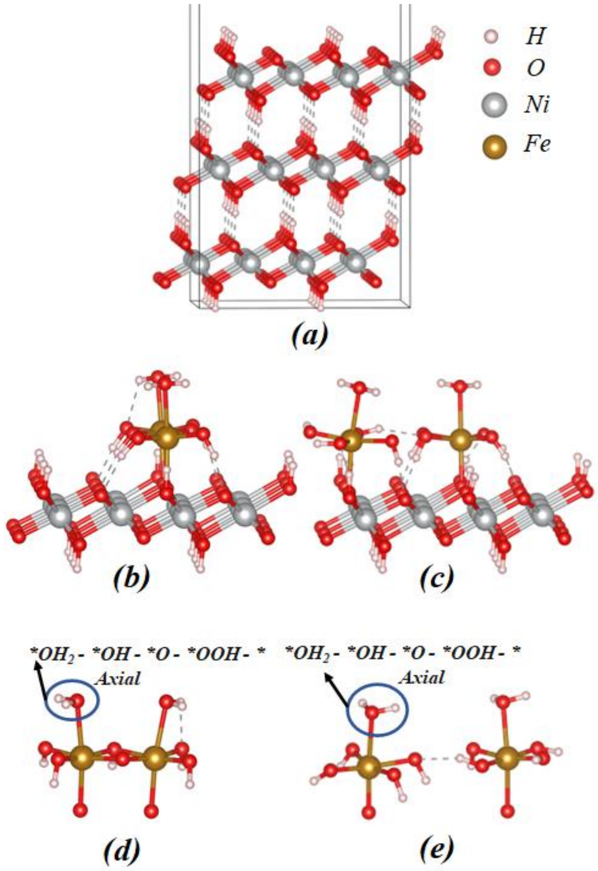

Supplementary Figure 31 | (a) The structure of bare NiOOH (001) surface. The adsorption model for the **(b)** Fe-O-Fe dimer and **(c) isolated** Fe-O on the NiOOH (001) surface. Reaction sites (circled in blue), Fe-coordination and reaction pathways with intermediates involved in the OER process for the **(d)** Fe-O-Fe dimer and **(e) isolated** Fe-O case. Only the upper part of the (001) surface is shown for clarity.

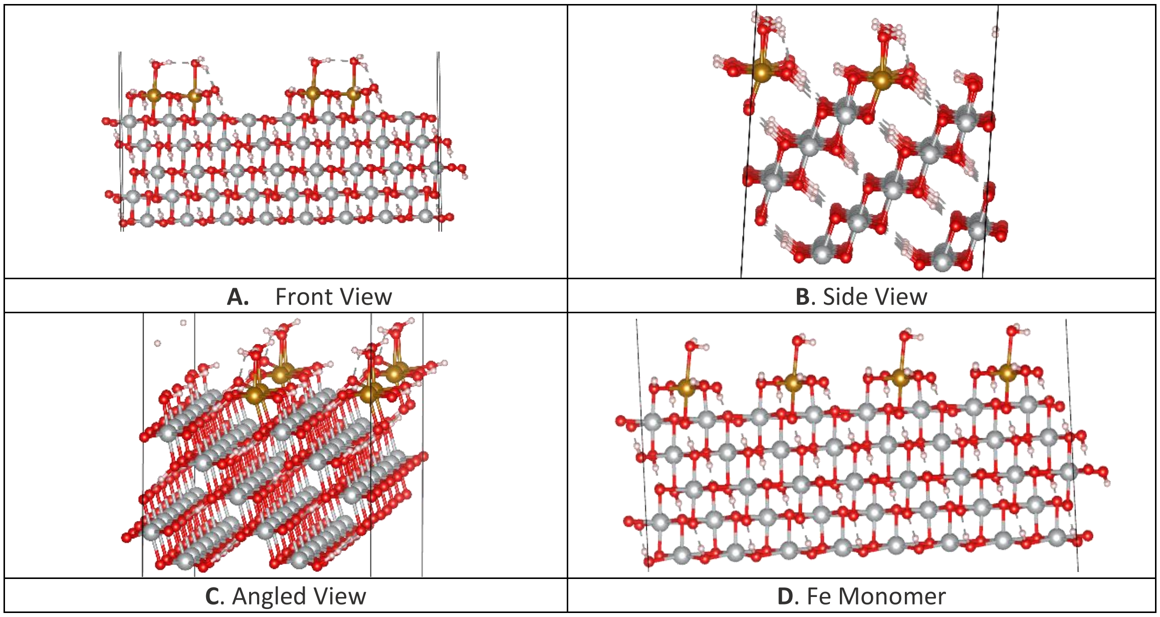

Supplementary Figure 32 | The different angular view of the adsorption model for the Fe-O-Fe dimer clusters **(a)** The front view **(b)** side view **(c)** angular view. **(d)** The front view of the adsorption model for the Fe monomer clusters. The Fe dimer cluster on the NiOOH edge/surface is distinct from a Ni vacancy. To test whether an Fe adsorbed dimer cluster facilitates OER, we have considered an Fe cluster where Fe has four OH bonds in plane parallel to the oxygen atoms of the NiOOH (monomer) and for the dimer where two Fe atoms are bonded to the NiOOH slab and to each other via a bridging oxygen and terminated otherwise with 4 OH bonds. The rest of the coordination is satisfied by one water molecule on top. Hence, the adsorbed cluster has a different structure and symmetry from possible vacancies on NiOOH. All Ni atoms on the surface of the dimer model are bonded to -OH if they are not attached to Fe atoms.

Supplementary Table 4. Supporting numerical data.

Sample Identification	Deposition parameters	Mass Ni[a] (ng cm^{-2})	Mass Co[a] (ng cm^{-2})	Mass Fe[a] (ng cm^{-2})	Fe/Ni at. ratio (%)	Fe/Co at. ratio (%)	TOF_{Fe} (s^{-1}) η = 300 mV	TOF_{Fe} (s^{-1}) η = 350 mV
NiO_xH_y	0.1 M Ni^{2+} -0.1 mA cm^{-2} 120 s	2744 ± 43	—	—	—	—	—	—
CoO_xH_y	0.1 M Co^{2+} -2 mA cm^{-2} 8 s	—	4966 ± 213	—	—	—	—	—
Fe incorporated NiO_xH_y from CA Fe-spiking		2713 ± 47	—	19 ± 2	0.7 ± 0.3	—	1.9 ± 0.3	4.5 ± 0.8
Fe incorporated NiO_xH_y from CA Fe-spiking		2941 ± 58	—	45 ± 8	1.6 ± 0.3	—	3.1 ± 0.7	13.5 ± 3.2
Fe incorporated NiO_xH_y from CA Fe-spiking	0.1 M Ni^{2+} -0.1 mA cm^{-2} 120 s	2737 ± 92	—	53 ± 7	2.0 ± 0.4	—	4.3 ± 0.6	22.3 ± 1.0
Fe incorporated NiO_xH_y from CA Fe-spiking		2843 ± 56	—	77 ± 11	2.9 ± 0.4	—	5.8 ± 0.8	28.4 ± 1.9
Fe incorporated NiO_xH_y from CA Fe-spiking		2956 ±144	—	87 ± 5	3.1 ± 0.5	—	8.4 ± 0.4	35.5 ± 3.7

Fe incorporated NiO_xH_y from CA Fe-spiking	0.1 M Ni^{2+} -0.1 mA cm^{-2} 120 s	2975 ± 122	—	144 ± 19	5.1 ± 0.5	—	10.4 ± 1.4	39.6 ± 1.9
Fe incorporated NiO_xH_y from CA-2CV Fe-spiking		2636 ± 22	—	207 ± 11	8.3 ± 0.4	—	6.7 ± 0.2	28.2 ± 1.0
Fe incorporated NiO_xH_y from CA-5CV Fe-spiking		3033 ± 34	—	337 ± 16	11.7 ± 0.4	—	4.3 ± 0.2	24.3 ± 1.7
Fe incorporated NiO_xH_y from CA-10CV Fe-spiking		2853 ± 47	—	389 ± 31	13.9 ± 1.2	—	3.7 ± 0.2	18.9 ± 0.4
Fe incorporated NiO_xH_y from CA-20CV Fe-spiking		2790 ± 27	—	508 ± 16	19.0 ± 0.5	—	3.5 ± 0.2	16.3 ± 0.6
Co-deposited $Ni_{0.95}Fe_{0.05}O_xH_y$	0.09975 M Ni^{2+} 0.00025 M Fe^{2+} -0.1 mA cm^{-2} 120 s	2929 ± 55	—	136 ± 17	4.9 ± 0.4	—	5.2 ± 0.2	25.5 ± 3.3
Co-deposited $Ni_{0.93}Fe_{0.07}O_xH_y$	0.0995 M Ni^{2+} 0.0005 M Fe^{2+} -0.1 mA cm^{-2} 120 s	2868 ± 42	—	194 ± 13	7.1 ± 0.5	—	4.4 ± 0.2	23.4 ± 4.0
Co-deposited $Ni_{0.91}Fe_{0.09}O_xH_y$	0.099 M Ni^{2+} 0.001 M Fe^{2+} -0.1 mA cm^{-2} 120 s	2842 ± 153	—	252 ± 27	9.3 ± 1.2	—	3.3 ± 0.2	16.7 ± 2.2
Co-deposited $Ni_{0.84}Fe_{0.16}O_xH_y$	0.098 M Ni^{2+} 0.002 M Fe^{2+} -0.1 mA cm^{-2} 120 s	2737 ± 66	—	500 ± 10	19.2 ± 1.1	—	3.0 ± 0.1	13.4 ± 0.9
Fe incorporated CoO_xH_y from CA Fe-spiking	0.1 M Co^{2+} -2 mA cm^{-2} 8 s	—	4859 ± 306	40 ± 8	—	0.9 ± 0.1	0.27 ± 0.05	4.3 ± 0.2
Fe incorporated CoO_xH_y from CA Fe-spiking		—	5284 ± 356	64 ± 8	—	1.3 ± 0.2	0.35 ± 0.05	4.7 ± 0.7
Fe incorporated CoO_xH_y from CA Fe-spiking	0.1 M Co^{2+} -2 mA cm^{-2} 8 s	—	4988 ± 382	81 ± 9	—	1.7 ± 0.3	0.54 ± 0.12	6.2 ± 0.6
Fe incorporated CoO_xH_y from CA Fe-spiking		—	4951 ± 296	114 ± 12	—	2.4 ± 0.3	0.71 ± 0.08	7.1 ± 0.8
Co-deposited $Co_{0.98}Fe_{0.02}O_xH_y$	0.099 M Co^{2+} 0.001 Fe^{2+} -2 mA cm^{-2} 8 s	—	4992 ± 119	111 ± 16	—	2.3 ± 0.3	0.23 ± 0.06	4.0 ± 0.4
Co-deposited $Co_{0.97}Fe_{0.03}O_xH_y$	0.098 M Co^{2+} 0.002 Fe^{2+} -2 mA cm^{-2} 8 s	—	5108 ± 231	167 ± 13	—	3.5 ± 0.2	0.23 ± 0.04	3.6 ± 0.2
Co-deposited $Co_{0.94}Fe_{0.06}O_xH_y$	0.096 M Co^{2+} 0.003 Fe^{2+} -2 mA cm^{-2} 8 s	—	4724 ± 142	264 ± 15	—	5.9 ± 0.4	0.18 ± 0.02	2.5 ± 0.3
Co-deposited $Co_{0.92}Fe_{0.08}O_xH_y$	0.093 M Co^{2+} 0.007 Fe^{2+} -2 mA cm^{-2} 8 s	—	4258 ± 83	365 ± 18	—	9.0 ± 0.4	0.14 ± 0.01	2.2 ± 0.1

[a] Mass of metals was determined by ICP-MS analysis at least in triplicate.

Supplementary Table 5. Additional activity metric summary of investigated samples.

Sample Identification	*J* @ η = 300 mV (mA cm^{-2})	*J* @ η = 350 mV (mA cm^{-2})	η @ 10 mA cm^{-2} (mV)
Fe-incorporated NiO_xH_y from CA Fe spiking – 1 CV	4.0	11.4	340
Fe-incorporated NiO_xH_y from CA Fe spiking – 10 CV	5.1	12.4	335
Fe-incorporated CoO_xH_y from CA Fe spiking – 1 CV	0.14	1.7	n/a*
Fe-incorporated CoO_xH_y from CA Fe spiking – 10 CV	0.16	2.1	n/a*

*indicates experiments were not performed at or above 10 mA cm^{-2}

Supplementary Table 6: Oxidation states for Fe-O monomer and Fe-O-Fe dimer. The respective Ni substitution cases for all are as mentioned in Figure 5 of the manuscript with the mechanisms given above. M represents the magmom value in the output file and O represents the corresponding oxidation state.*

Reaction Name	PDS		Atom Name	Before		After	
				M	O	M	O
Fe-O monomer	OOH	OO	Fe	4.17	3	3.60	4
			Fe	3.75	3	3.72	3
Fe-O monomer (Ni subs)	OH	O	Ni	1.19	3	0.07	4
			Fe	3.06	5	2.41	6
Fe-O-Fe Axial	H_2O	OH	Ni	1.39	3	1.47	2
			Ni	1.39	3	1.73	2
			Fe	4.18	3	3.72	4
			Fe	3.85	3	3.60	4
Fe-O-Fe Insertion	H_2O	OH	Ni	1.39	3	1.47	2
			Ni	1.39	3	1.73	2
			Fe	4.18	3	3.72	4
			Fe	3.85	3	3.60	4
Fe-O-Fe Bridge	OH	O	Ni	1.12	3	0.10	4
			Fe	4.22	3	3.85	4
Fe-O-Fe Equatorial	OH	O	Ni	1.39	3	0.50	4
			Ni	1.31	3	1.42	2
			Fe	3.80	3	3.66	4
			Fe	3.88	3	3.75	4

Fe-O-Ni Axial	OH	O	Fe	3.62	4	2.58	5
Fe-O-Ni Insertion	H_2O	OH	Fe	3.97	3	3.62	4
Fe-O-Ni Bridge	OH	O	Ni	1.40	2	1.36	3
			Ni	1.00	3	0.06	4
			Ni	1.31	3	1.40	2
Fe-O-Ni Equatorial	OH	O	Ni	1.34	3	1.42	2
			Fe	3.63	2	3.65	4
Fe-O monomer (001)	OH	O	Fe	3.62	3	3.62	3
			Fe	3.10	4	2.91	5
Fe-O-Fe Axial (001)	H_2O	OH	Fe	3.66	3	3.17	4
			Fe	3.69	3	3.11	4

*According to previous work[14, 15], the magnetization range 3.75 to 4.3 is assigned to +3 oxidation state and the magnetization range 3.2 to 3.75 is assigned to +4 or +2 oxidation states, depending on the mechanism, while the values ~3.6-3.8 are on the boarder of these two ranges. In cases where there is a change in the magnetization within the range, but the change is significant, we regard this as a change in the oxidation state. Note that in some cases there is a partial change in charge and not a full change in oxidation state as indicated, depending on the magnitude of the magnetic moment change. However, we indicated the best estimate to emphasize that there is a change in atomic charges.

Supplementary Table 7. Free energies and theoretical overpotentials from Table 3 plus those calculated for a "core" Fe active site, an Fe that is substituted at a Ni position according to the model below.

Gibbs free energy change	$(0\bar{1}5)$ surface														
	isolated Fe-O			Fe-O-M dimer											
	Axial			axial			insertion			bridge			equatorial		
	Fe	Ni	Core	Fe	Ni	Core	Fe	Ni	Core	Fe	Ni	Core	Fe	Ni	Core
ΔG_1	1.60	1.62	1.52	1.73	1.80	2.04	1.73	1.80	2.04	1.45	1.41	1.59	1.16	1.48	1.44
ΔG_2	2.03	2.03	2.34	1.67	2.12	1.82	0.93	1.01	0.74	1.56	1.78	1.95	1.70	1.92	1.74
ΔG_3	-1.16	1.03	-1.12	1.23	0.65	0.68	1.59	1.53	2.12	1.05	0.96	0.71	0.52	0.27	-0.02
ΔG_4	2.19	-0.03	1.85	0.22	0.36	0.44	0.67	0.58	0.03	0.86	0.76	0.67	1.47	1.46	1.49
ΔG_5	0.26	0.27	0.34	0.07	0.00	-0.06	–	–	–	–	–		0.07	-0.20	0.27
η_{th}	0.96	0.80	1.11	0.50	0.89	0.81	0.50	0.57	0.89	0.33	0.55	0.72	0.47	0.68	0.51

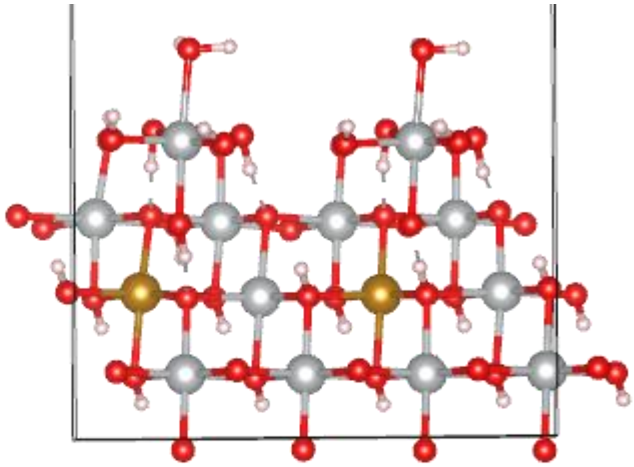

Supplementary Figure 33 | The slab model used to calculate the “core” Fe Gibbs free energies in Table 6 above. Gray spheres are Ni, red spheres are oxygen, and gold spheres are Fe atoms.

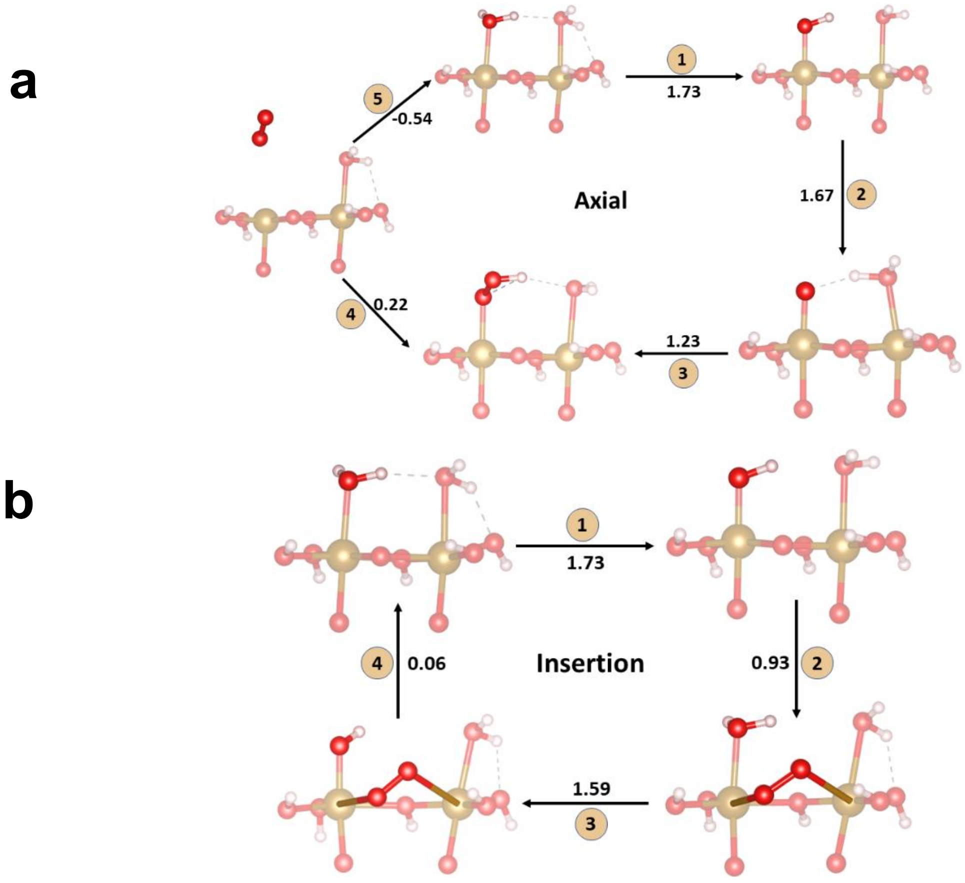

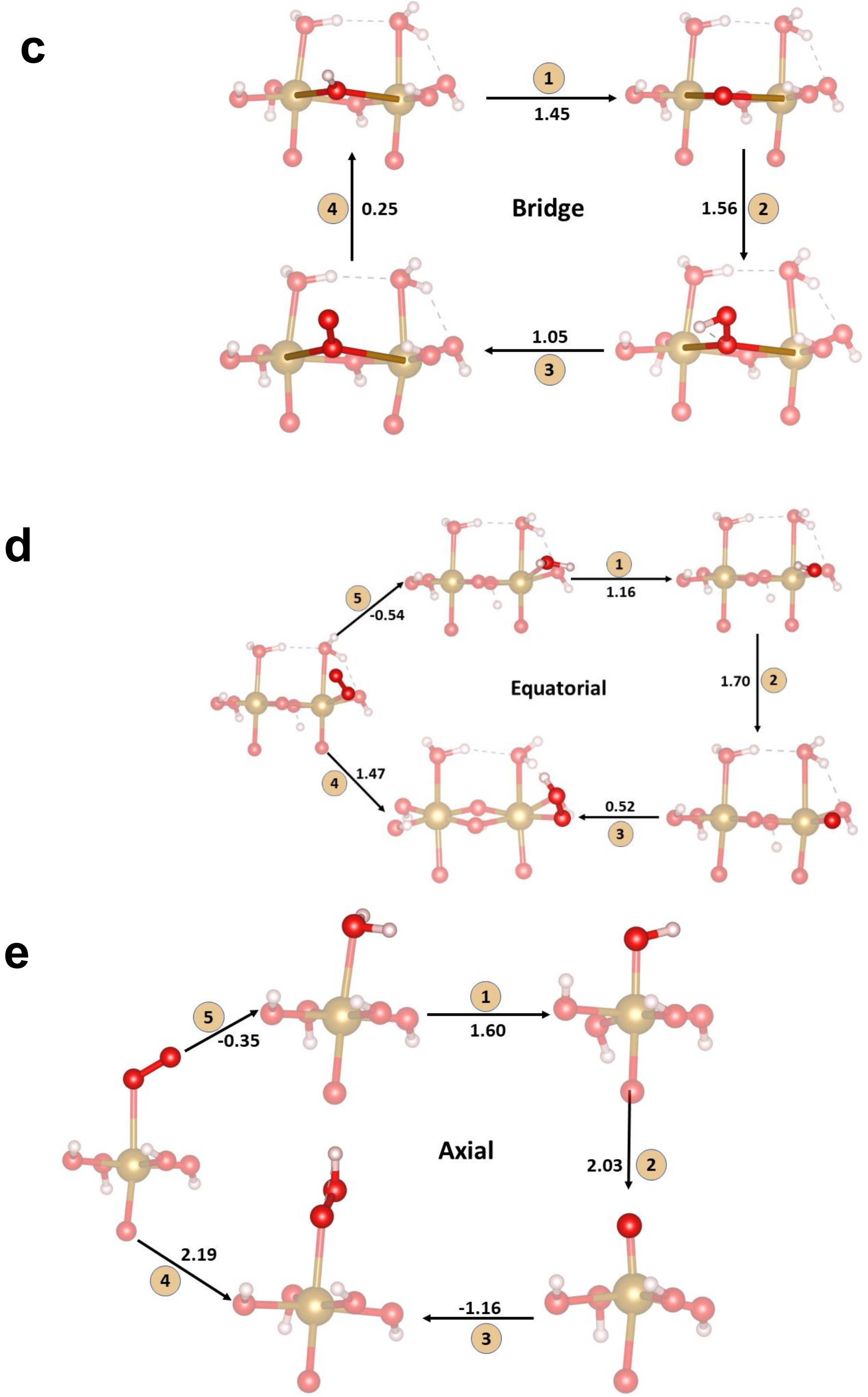

Supplementary Figure 34 |Additional large-scale depictions of dimer and monomer reaction pathways used in DFT calculations: (**a-d)** dimer mechanisms and **(e)** monomer mechanism.

Supplementary References

1. Zizak I. The KMC-3 XPP beamline at BESSY II. *J. Large-Scale Res. Fac.*; 2017.

2. Schuck G, Zisak I. CryoEXAFS: X-ray absorption spectroscopy station with cryogenic or in-beam operando electrochemistry sample conditions at BESSY II. *J large-scale res fac* 2020, **6**.

3. Bearden JA, Burr AF. Reevaluation of X-Ray Atomic Energy Levels. *Reviews of Modern Physics* 1967, **39**(1)**:** 125-142.

4. Villalobos J, Golnak R, Xi L, Schuck G, Risch M. Reversible and irreversible processes during cyclic voltammetry of an electrodeposited manganese oxide as catalyst for the oxygen evolution reaction. *Journal of Physics: Energy* 2020, **2**(3)**:** 034009.

5. Ankudinov AL, Ravel B, Rehr JJ, Conradson SD. Real-space multiple-scattering calculation and interpretation of x-ray-absorption near-edge structure. *Physical Review B* 1998, **58**(12)**:** 7565-7576.

6. Oleś A, Szytuła A, Wanic A. Neutron Diffraction Study of γ-FeOOH. *physica status solidi (b)* 1970, **41**(1)**:** 173-177.

7. Risch M, Klingan K, Ringleb F, Chernev P, Zaharieva I, Fischer A*, et al.* Water Oxidation by Electrodeposited Cobalt Oxides—Role of Anions and Redox-Inert Cations in Structure and Function of the Amorphous Catalyst. *ChemSusChem* 2012, **5**(3)**:** 542-549.

8. Hunter BM, Thompson NB, Müller AM, Rossman GR, Hill MG, Winkler JR*, et al.* Trapping an Iron(VI) Water-Splitting Intermediate in Nonaqueous Media. *Joule* 2018, **2**(4)**:** 747-763.

9. Burke MS, Zou S, Enman LJ, Kellon JE, Gabor CA, Pledger E*, et al.* Revised Oxygen Evolution Reaction Activity Trends for First-Row Transition-Metal (Oxy)hydroxides in Alkaline Media. *The Journal of Physical Chemistry Letters* 2015, **6**(18)**:** 3737-3742.

10. Zou S, Burke MS, Kast MG, Fan J, Danilovic N, Boettcher SW. Fe (oxy)hydroxide oxygen evolution reaction electrocatalysis: Intrinsic activity and the roles of electrical conductivity, substrate, and dissolution. *Chem Mater* 2015, **27:** 8011-8020.

11. Dette C, Hurst MR, Deng J, Nellist MR, Boettcher SW. Structural Evolution of Metal (Oxy)hydroxide Nanosheets during the Oxygen Evolution Reaction. *ACS Applied Materials & Interfaces* 2019, **11**(6)**:** 5590-5594.

12. Subbaraman R, Tripkovic D, Chang K-C, Strmcnik D, Paulikas AP, Hirunsit P*, et al.* Trends in activity for the water electrolyser reactions on 3d M(Ni,Co,Fe,Mn) hydr(oxy)oxide catalysts. *Nature Materials* 2012, **11**(6)**:** 550-557.

13. Rumble JR, *CRC Handbook of Chemistry and Physics, 103rd Edition*. CRC Press LLC, 2021.

14. Zaffran J, Burke-Stevens M, Trang CDM, Nagli M, Shehadeh M, Boettcher SW*, et al.* Influence of electrolyte cations on Ni(Fe)OOH Catalyzed Oxygen Evolution Reaction. 2017, **29:** 4761-4767.

15. Fidelsky V, Toroker MC. The secret behind the success of doping nickel oxyhydroxide with iron. *Physical Chemistry Chemical Physics* 2017, **19**(11)**:** 7491-7497.
